# Basal cytoplasmic calcium levels regulate *C. elegans* germ stem cell proliferation

**DOI:** 10.1242/dev.205301

**Published:** 2026-07-08

**Authors:** Fariha J. Khan, Parva M. Vyas, Aahana N. Shankaran, Alexandra C. Wells, Corrissa J. Velder, Rabia N. Kaya, Anuj R. Nagula, Edward T. Kipreos

**Affiliations:** Department of Cellular Biology, University of Georgia, Athens, GA 30602, USA

**Keywords:** Basal calcium, Germ cells, Cell proliferation, Germline development, Sex determination

## Abstract

The *Caenorhabditis elegans* germline is one of the foremost models for the study of germ cell biology. We created an integrated ratiometric calcium reporter to assess calcium levels in the *C. elegans* germ line. The reporter reveals changes in basal cytoplasmic calcium levels during hermaphrodite sexual development. Oogenic cells have elevated calcium levels, while spermatogenic cells have significantly reduced levels of calcium. There are a small number of germ cells in the bend region of mid-to-late L4 hermaphrodite germ lines with elevated calcium levels that are correlated with the spermatogenesis-to-oogenesis transition. We identified GON-2 as a calcium channel that is required for the maintenance of normal calcium levels in the germline. Significantly, basal calcium levels correlate with germ cell proliferation. Starvation and inactivation of pro-proliferation signaling pathways reduce basal calcium levels, while proliferating germline tumor cells have elevated basal calcium levels. Experimentally altering calcium levels leads to a concomitant increase or decrease in the proliferation rate of germ stem cells. These results imply that basal cytoplasmic calcium acts as a rheostat to regulate germ stem cell proliferation.

## Teaser

**Summary:** A new ratiometric Ca^2+^ reporter reveals dynamic changes in cytoplasmic Ca^2+^ during *C. elegans* germline sexual development and identifies basal Ca^2+^ levels as a regulator of germ stem cell proliferation.

## INTRODUCTION

**Fig. 1.**
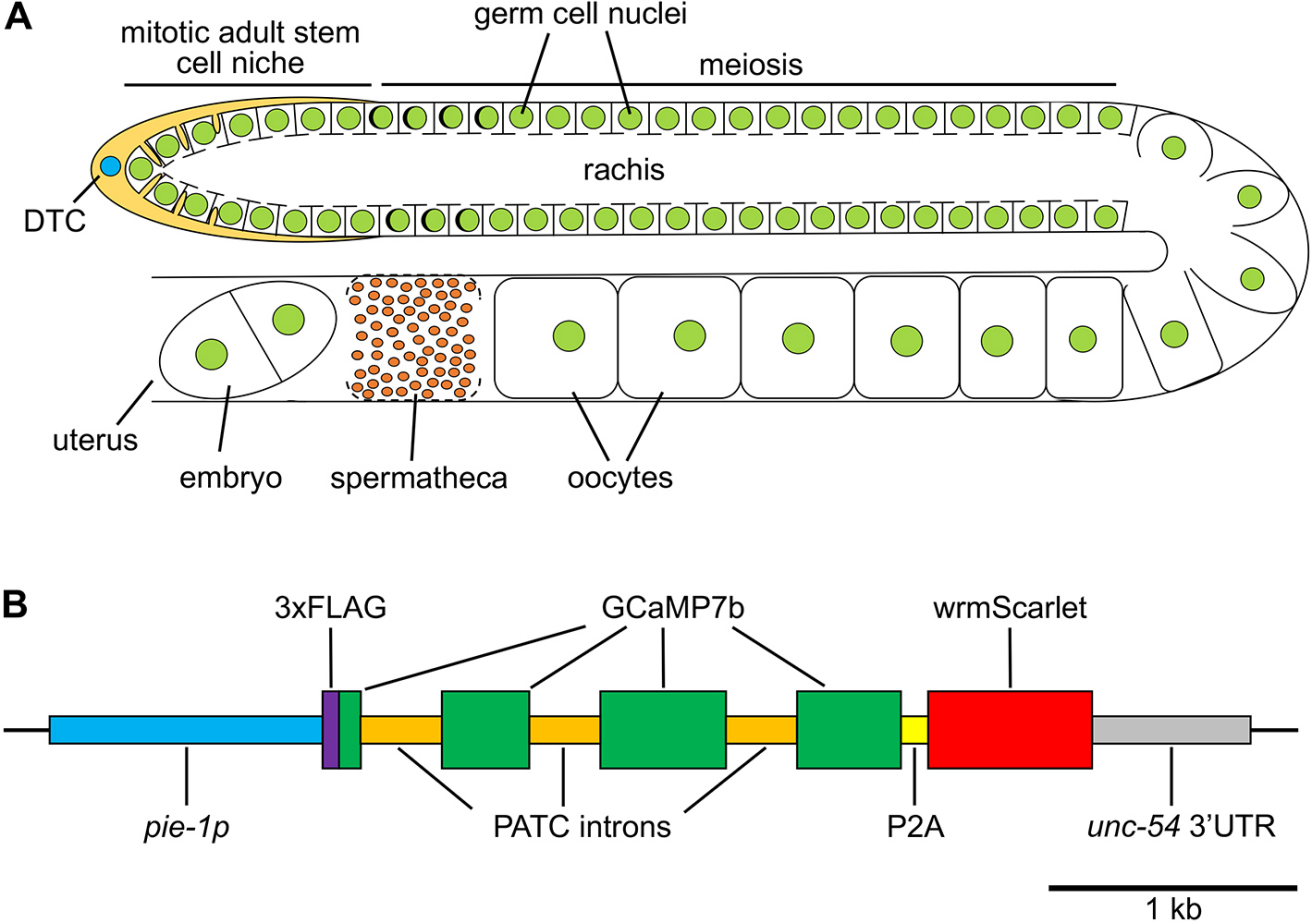
**Diagrams of a *C. elegans* adult hermaphrodite gonad arm and the GCaMP7b::wrmScarlet genomic insertion.** (A) A mid-section view of one of the two U-shaped gonad arms in an adult hermaphrodite. The distal tip cell (DTC) is a somatic gonadal cell that creates the mitotic adult stem cell niche. Germ cell nuclei are in green. Note the opening of germ cells to the common cytoplasm, termed the rachis. Sperm are shown in red within the spermatheca somatic gonadal structure. (B) Diagram of the *pie-1p*::3xFLAG::GCaMP7b::P2A::wrmScarlet transgenic insertion in the SKI LODGE locus on chromosome III.

The *Caenorhabditis elegans* germ line is a model system for studying germ cell biology (Hubbard and Schedl, 2019). The hermaphrodite germ line has two U-shaped arms, and contains both male and female germ cells and associated somatic gonad structures (Fig. 1A). The hermaphrodite gonad switches from male (sperm producing) to female (oocyte producing) states during the L4 larval and young adult stages. Sperm are stored in the spermatheca, a somatic gonadal structure between the uterus and the oviduct in the proximal gonad.

In older adult hermaphrodites, the mitotic proliferation of germ stem cells is confined to the most distal region of the germ line in an adult stem cell niche defined by the projections of the distal tip cell (DTC) (Fig. 1A) (Byrd et al., 2014). The DTC signals via GLP-1/Notch to maintain germ cells in a mitotic state. In older adult hermaphrodites, as germ cells leave the DTC-defined stem cell niche, reduced Notch signaling and the action of pro-meiotic RNA regulatory pathways promote meiotic entry and differentiation (Albarqi and Ryder, 2022; Hubbard and Schedl, 2019). Once germ cells exit the DTC-defined stem cell niche, they enter meiosis and will begin the regulatory program for oocyte differentiation.

The *C. elegans* germ line is a partial syncytium in which germ cells are connected to a common cytoplasm through openings in their plasma membrane (Zellag et al., 2025) (Fig. 1A). This partial syncytium allows the germ cells to produce maternal product (mRNA and protein) that is transferred to the developing oocytes to allow rapid cell divisions in the early embryo. There does not appear to be mixing of cytoplasm between germ cells because mitosis is not synchronous, whereas it would be synchronous if activated mitotic cyclin/CDK complexes transferred between mitotic and non-mitotic cells (Kipreos, 2005).

In the wild, many *C. elegans* spend much of their lives searching for bacteria (their food source) in a nutrient-deprived state (Frézal and Félix, 2015). When food-deprived *C. elegans* hermaphrodites encounter rotting vegetation or organic matter full of bacteria, they respond by increasing their rate of germ cell proliferation to rapidly produce progeny (Korta et al., 2012; Roy et al., 2016; Seidel and Kimble, 2015). An adult nematode can produce ∼300 progeny in 3 days, and each of those progenies can produce ∼300 progeny in ∼6 days. Thus, any food source, no matter how abundant, is quickly depleted. In experimental settings that recapitulate a boom–bust life cycle, nematodes that can more rapidly produce progeny have an advantage over nematodes that produce more progeny over a longer period of time (Hodgkin and Barnes, 1991).

The rate of generating germ cells in the mitotic zone is modulated by signaling pathways in response to environmental conditions (including the presence or absence of food). Germ cell proliferation in larvae is promoted by insulin signaling via specific insulin ligands (INS-3 and INS-33), as well as cell-intrinsic assessment of nutrient availability that involves S6 kinase and the TOR/RAPTOR pathway (Korta et al., 2012; Michaelson et al., 2010). Loss of these signaling pathways causes a decrease in germ cell proliferation in larvae. TGF-β signaling is thought to promote mitotic germ cell numbers primarily by functioning in the DTC to promote the expression of the LAG-2 ligand that signals through the GLP-1 Notch receptor on germ cells to prevent mitotic germ cells from entering meiosis (Dalfó et al., 2012; Pekar et al., 2017). However, TGF-β signaling can promote germ cell proliferation independently of GLP-1, as shown by loss of TGF-β signaling reducing the proliferation of germ cell tumors lacking *glp-1* (Dalfó et al., 2012). Dietary restriction or short-term food deprivation also decreases germ cell proliferation, while extended starvation causes a cessation of proliferation (Korta et al., 2012; Roy et al., 2016; Seidel and Kimble, 2015).

In the *C. elegans* hermaphrodite gonad, calcium (Ca^2+^) plays several roles associated with fertilization and the initiation of embryogenesis. Major sperm protein (MSP), secreted by sperm, binds receptors on the proximal oocyte, including the Eph receptor VAB-1, and promotes meiotic maturation via Ca^2+^-dependent pathways, including calmodulin-dependent protein kinase II (Corrigan et al., 2005). Fertilization of an oocyte in the spermatheca initiates a wave of Ca^2+^ at the site of sperm entry that propagates across the oocyte (Takayama and Onami, 2016). This Ca^2+^ wave prevents polyspermy and initiates embryogenesis, and is present during egg activation for all animal species examined (Stein et al., 2020). Finally, oocyte entry into the spermatheca initiates a series of inositol 1,4,5-trisphosphate (IP3)-dependent Ca^2+^ oscillations in the spermatheca that lead to constrictions that move the zygote into the uterus (Kovacevic et al., 2013).

Here, we report the use of a ratiometric Ca^2+^ reporter to allow measurements of basal levels of cytoplasmic Ca^2+^ in the germline. To date, changes in Ca^2+^ levels have not been linked to proliferation in *C. elegans*. We show that the basal level of cytoplasmic Ca^2+^ correlates with the proliferation rate of mitotic germ cells. Moreover, altering basal Ca^2+^ levels alters the proliferation rate of germ cells, indicating that basal Ca^2+^ levels can regulate proliferation. Additionally, we identify a calcium channel required for maintaining basal cytoplasmic Ca^2+^ levels, and identify changes in cytoplasmic Ca^2+^ levels associated with the development of oocytes and sperm.

## RESULTS

### A ratiometric germline cytoplasmic Ca^2+^ reporter

In order to assess the relative levels of Ca^2+^ in the *C. elegans* germ line, we created an integrated ratiometric Ca^2+^ reporter that is expressed in germ cells. The reporter comprises the GCaMP7b genetic calcium reporter (a modified GFP that only fluoresces when bound to Ca^2+^; Dana et al., 2019) followed by a P2A ribosome skipping sequence and wrmScarlet (a *C. elegans*-codon-optimized mScarlet; El Mouridi et al., 2017) (Fig. 1B). During translation, the newly synthesized GCaMP7b protein is released from the ribosome as it encounters the P2A ribosome skipping sequence, and then the ribosome translates a separate wrmScarlet protein. Thus, there are equal translations of GCaMP7b and wrmScarlet. Because there should be a one-to-one ratio of GCaMP7b and wrmScarlet, the ratio of GCaMP7b fluorescence divided by wrmScarlet fluorescence provides a ratiometric assessment of Ca^2+^ levels that is normalized for differences in reporter expression. To ensure that conditions of the microscope (e.g. confocal laser strength) did not affect quantitative comparisons, we analyzed control and experimental samples at the same microscope sessions and standardized the experimental results relative to the control samples.

To generate the ratiometric Ca^2+^ reporter, we utilized the SKI LODGE system in which tissue-specific reporters are placed in the genome upstream of a CRISPR targeting sequence from the *dpy-10* gene (Silva-Garcia et al., 2019). We used two rounds of CRISPR/Cas9 genome editing to place GCaMP7b and the P2A::wrmScarlet cassette into the SKI LODGE site on chromosome III that contains the germline-specific promoter *pie-1p*. The GCaMP7b sequence was modified by optimizing the codons for *C. elegans* and introducing three PATC-enriched introns, which promote expression of transgenes in the germ line (Aljohani et al., 2020) (Fig. 1B). The transgene is referred to here as pGCS (for *pie-1p*::GCaMP7::P2A::wrmScarlet).

### Ca^2+^ levels are altered during the sexual development of the hermaphrodite germ line

**Fig. 2.**
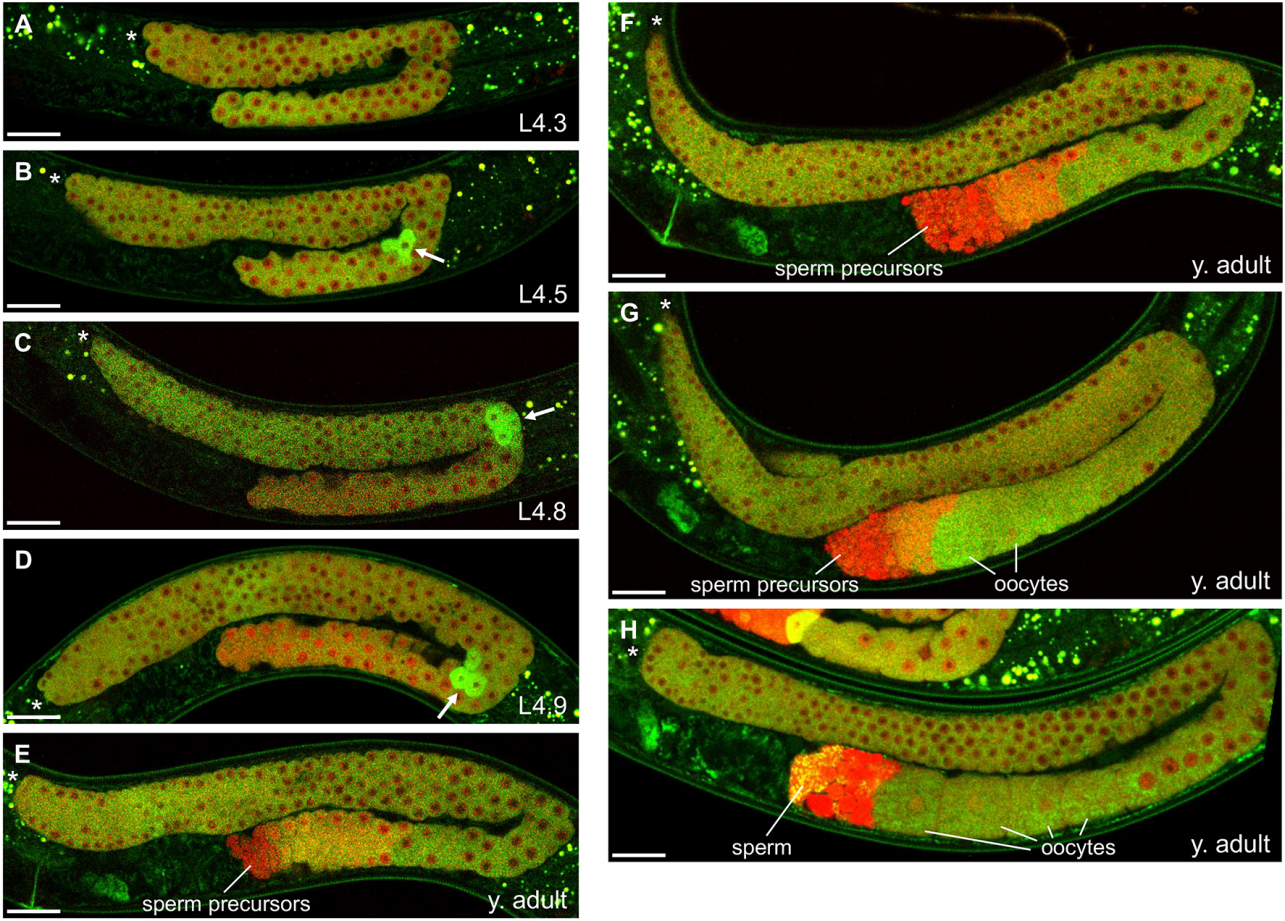
**Ca^2+^ levels during hermaphrodite sexual development in L4 and young adult stages.** (A-H) GCaMP7b (green) and wrmScarlet (red) overlay of confocal images of gonad arms from hermaphrodites of the indicated ages, presented from younger (A) to older (H) animals. L4-stage larvae are categorized by their sub-stages, which are based on vulval morphology. Young adults (without eggs) are ordered based on the development of their oocyte region. Asterisks indicate the distal end of the gonad arm. Arrows indicate germ cells with high Ca^2+^ in the bend region of late L4 larvae. Oocytes and spermatogenic regions are marked. Small sections of D and H are cut off at the edge because the images were angled before cropping. Scale bars: 20 μm. Grayscale images for the two separate channels are shown in Fig. S1.

**Fig. 3.**
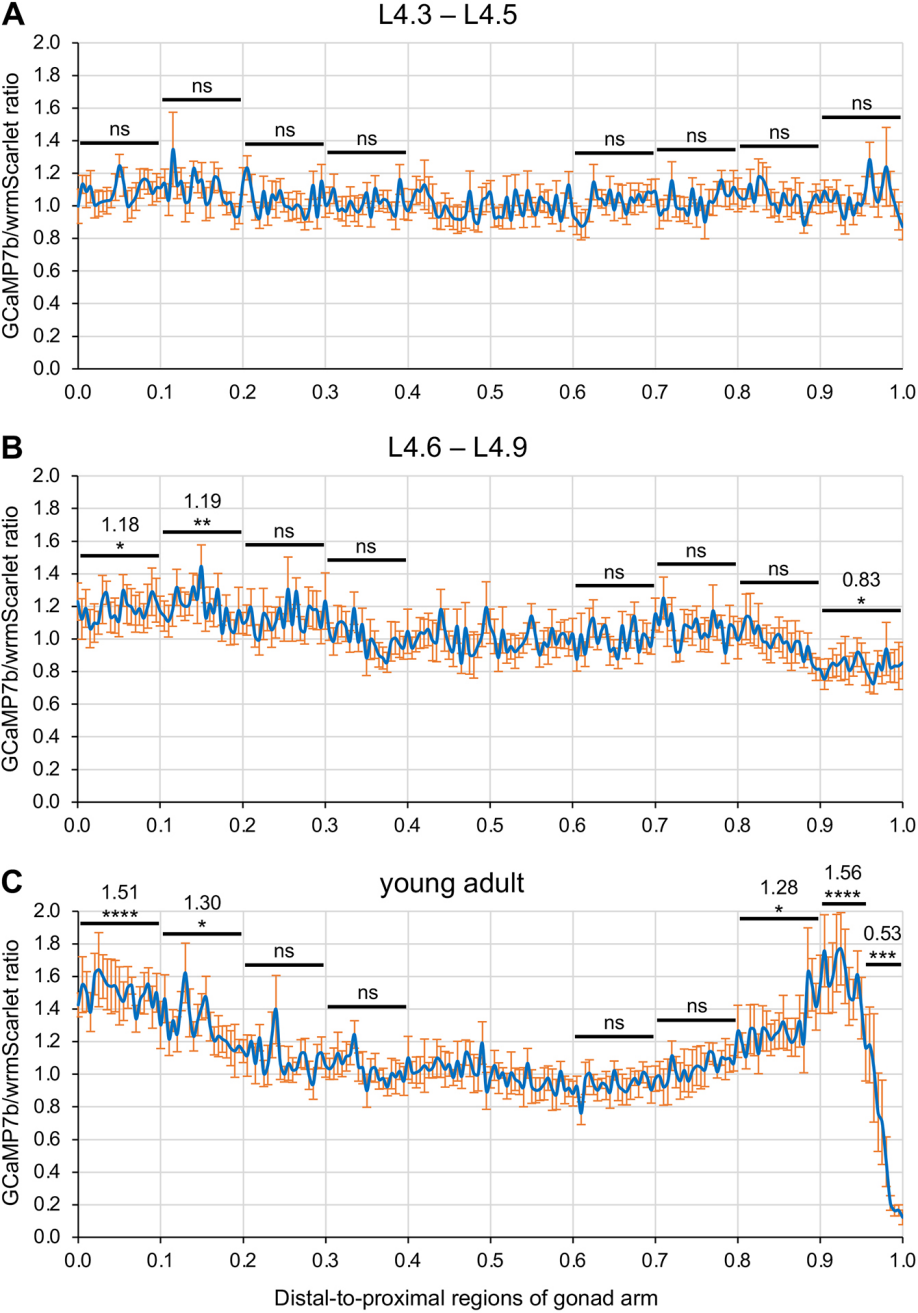
**Distal-to-proximal Ca^2+^ levels in L4 larval sub-stages.** (A-C) Line graphs of GCaMP7b/wrmScarlet signal distal to proximal for L4.3-L4.5 sub-stage larvae (A), L4.6-L4.9 (B) and older young adults (C). The line graphs represent the whole gonad arm (divided into 201 points) with data shown as mean±s.e.m. The distal end is at 0.0 and the proximal end is at 1.0. Each line graph is normalized so that the middle 20% (0.4-0.6) has an average value of 1.0. Statistical significance is calculated for deciles along the distal-to-proximal length relative to the middle 20%, with the ratio relative to the middle 20% provided for regions with significant differences. For C, the last two statistical comparisons cover the regions 0.90-0.96 and 0.96-1.0, with the regions chosen to reflect the differences in signal level. Statistical analysis was with one-way ANOVA with Dunnett's multiple comparisons. *P<0.05; **P<0.01; ***P<0.001; ****P<0.0001. ns, not significant. The numbers of gonad arms analyzed were *n*=15 (A), 12 (B) and 7 (C). The L4-stage gonad arms that were analyzed lacked elevated-Ca^2+^ cells to ensure that the signal from those cells did not impact the overall distal-to-proximal signal distribution.

The GCaMP7b expressed from the pGCS transgene appears to be largely excluded from nuclei and is therefore predominantly a cytoplasmic marker of Ca^2+^ levels (Fig. 2). The GCaMP7b/wrmScarlet ratio appears to be spatially uniform throughout the gonad from the L1 through the early L4 larval stages (Figs 2A,B and 3A; Fig. S1). The L4 larval stage can be subdivided into ten sub-stages (L4.0 to L4.9) based on the morphology of the vulva (Mok et al., 2015). In mid-to-late L4-stage larvae (L4.6 to L4.9), there is a modest increase (∼18%) in cytoplasmic Ca^2+^ levels in the distal region that includes the mitotic zone (Figs 2C,D and 3B; Fig. S1). This distal Ca^2+^ level increases in young adults (defined as adults without eggs), with Ca^2+^ levels ∼50% higher than the middle section of the gonad in older young adults (Figs 2E-H and 3C; Fig. S1).

The proximal region of the germline has a decrease in cytoplasmic Ca^2+^ levels in late L4-stage larvae that correlates with the beginning of spermatogenesis (Figs 2C,D and 3B; Fig. S1). In young adults, the level of Ca^2+^ progressively decreases as spermatogenesis progresses until the region with mature sperm is largely devoid of GCaMP7b signal (Figs 2E-H and 3C; Fig. S1). As the young-adult stage develops, the spermatogenic region with low Ca^2+^ levels appears to be pushed proximally by the developing oocytes with high Ca^2+^ levels (Figs 2E-H; Fig. S1).

In the later stages of spermatogenesis, there is an accumulation of wrmScarlet in the spermatogenic region coupled with a loss of GCaMP7b signal (Fig. S1). The most Ca^2+^-depleted objects in the spermatogenic region correspond to the residual bodies that remain after spermatid formation (Fig. 2H; Figs S1 and S2). Sperm also have reduced Ca^2+^ levels, but the levels are higher than for the residual bodies (Fig. S2).

### A small number of L4-stage germ cells have elevated Ca^2+^ levels

In the mid-to-late L4 stage, a small number of contiguous germ cells with high Ca^2+^ levels are often observed near the bend in the gonad arm (Fig. 2B-D; Fig. S1). In L4.3 and earlier sub-stages, we do not observe germ cells with elevated Ca^2+^ levels (Fig. S3A). In the L4.4 sub-stage, ∼40% of gonad arms exhibit cells with elevated Ca^2+^ (Fig. S3A). During the L4.5-L4.9 sub-stages, the percentage of gonad arms with elevated Ca^2+^-cells increases to ∼80%, with the observed range in the number of elevated Ca^2+^ cells per gonad arm varying from 1 to 22, with the mode being 5 cells per gonad arm (Fig. S3A). The GCaMP7b/wrmScarlet ratio is ∼2.7-fold higher in the elevated-Ca^2+^ cells relative to the cells surrounding them (Fig. S3B).

The cells with elevated Ca^2+^ alter their position relative to the bend during the L4 stage. We drew an imaginary line along the anterior-posterior axis through the midpoint of the bend of the gonad arm. During the L4.4 sub-stage, the elevated-Ca^2+^ cells are located proximal to this midpoint line, but during later L4 stages they become more evenly distributed around it (Fig. S3C).

Based on the timing and localization of the elevated Ca^2+^ cells, we considered whether there was a link to the spermatogenesis-to-oogenesis transition. The sperm-fate regulatory program begins in the L3 stage, whereas overt spermatogenic differentiation occurs during L4 (Ellis and Schedl, 2007; Lamont and Kimble, 2007; Noble et al., 2016). The temperature-sensitive period of gain-of-function mutations of *fem-3* (which block oogenesis) suggests that the oogenesis program initiates at least by late L4 stage (Barton et al., 1987). The appearance of the elevated-Ca^2+^ cells overlaps temporally with the spermatogenesis-to-oogenesis transition, as the L4.4 to L4.9 interval corresponds to the second half of the L4 stage (Mok et al., 2015). Further, the elevated-Ca^2+^ cells are localized to the bend region of the L4 gonad, which is between proximal spermatogenic cells and more distal cells that express an early marker of oogenesis (Ellis and Schedl, 2007).

To explore whether the sexual development of the germline affects the presence of the elevated-Ca^2+^ germ cells, we assessed their presence in different sex determination backgrounds. Loss-of-function Fem mutants feminize both the soma and the germline; Fem germlines fail to undergo spermatogenesis and instead produce only oocytes. Thus, both XX and XO Fem mutants develop as females. Fog mutants feminize just the germline, and so Fog XX mutants are females that produce only oocytes. Mog mutants masculinize the germline but do not alter the soma, and so Mog XX mutants have the somatic body plan of a hermaphrodite, but only produce sperm.

We observed that in both *fem-1*(*hc17ts*) and *fog-1*(*q325ts*) mutants at the non-permissive temperature of 25°C, no germ cells with elevated Ca^2+^ levels were observed in L4-stage germlines (Table 1). This suggests that the L4-stage elevated-Ca^2+^ cells require the presence of spermatogenesis. In two different Mog mutants, we observed a reduction in both the number of elevated Ca^2+^ cells per gonad arm and the percentage of gonad arms with elevated-Ca^2+^ cells (Table 1). This suggests that both spermatogenic and oogenic programs are required for the full manifestation of the elevated-Ca^2+^ cells.

**Table 1.** Elevated-Ca^2+^ cells in sex determination mutants

Genotype/conditions	Phenotype	Average elevated-Ca^2+^ cells/gonad arm	Kruskal–Wallis test versus WT XX	Gonad arms with elevated-Ca2^+^ cells (%)	Fisher's test versus WT XX	*n*
*fem-1*(*hc17ts*) XX at 25°C	Fem	0.0	****	0	****	12
*fog-1*(*q325ts*) XX	Fog	0.0	****	0	****	22
*fem-3*(*q96*) XX at 25°C	Mog	1.4	*	45	*	29
*mog-5*(*q449*) *unc-4*(*e120*) XX	Mog	0.41	****	28	****	29
wild type XX	Wild type	3.6	–	74	–	53
wild type XO	Wild type	0.13	****	6.3	****	16

*P<0.05; ****P<0.0001

### Reductions in germ cell proliferation reduce basal Ca^2+^ levels

We investigated whether conditions that reduce germ cell proliferation are correlated with changes in basal cytoplasmic Ca^2+^ levels in the germ line, particularly in the distal mitotic zone. We analyzed four conditions that are known to decrease mitotic germ cell proliferation.

**Fig. 4.**
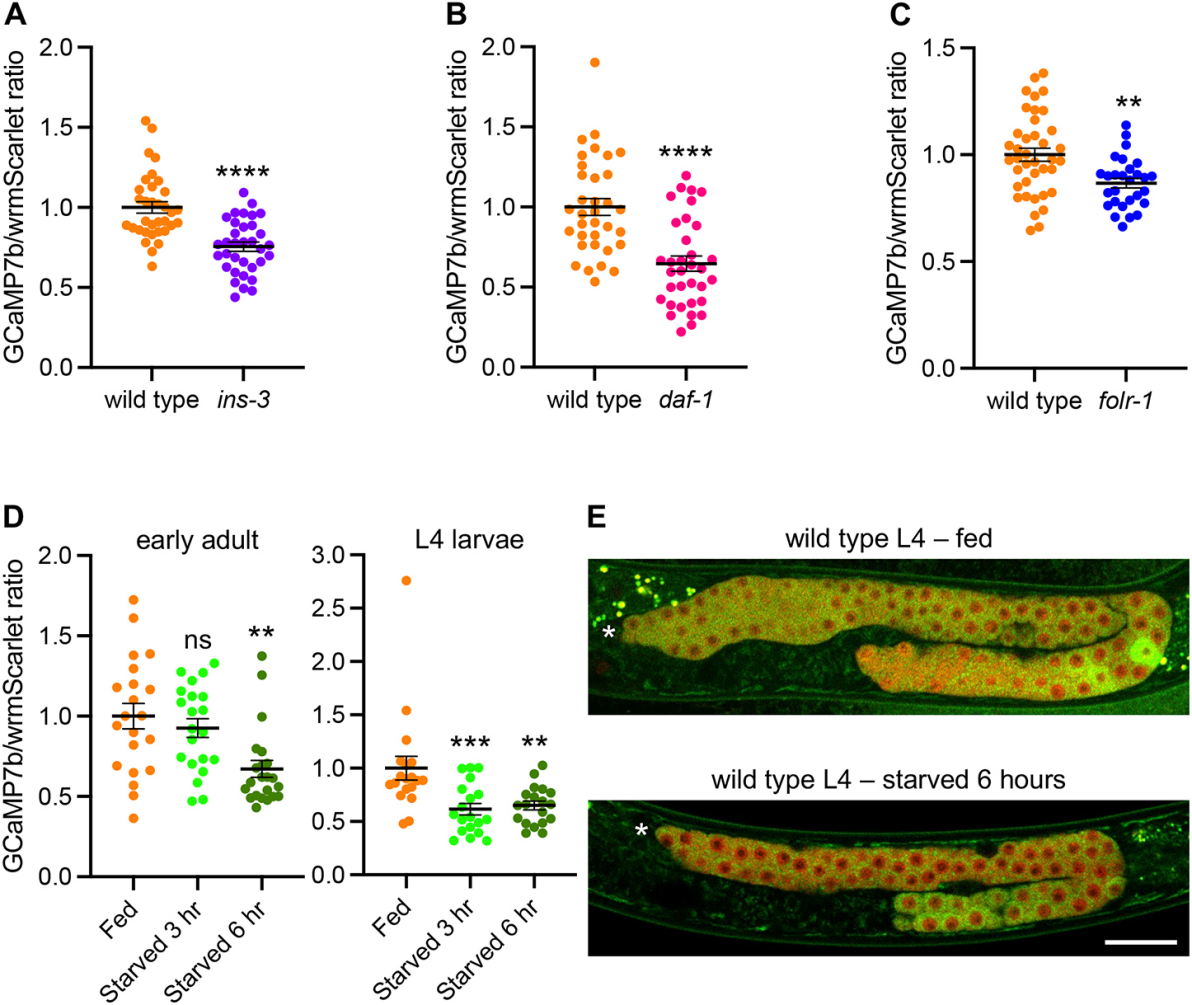
**Ca^2+^ levels in animals with reduced germ cell proliferation.** (A-C) Graphs of the GCaMP7b/wrmScarlet ratios in L4-stage *ins-3*(*ok2488*); pGCS (A), early adult *daf-1*(*m40ts*); pGCS at 25°C for 1 day (B), and L4-stage *folr-1*(*ek44*); pGCS (C) relative to comparably staged wild-type pGCS animals. (D) The GCaMP7b/wrmScarlet ratios in wild-type pGCS early adults (with one row of ∼6-10 eggs) or L4-stage larvae starved for 3 or 6 h, or fed. In all figures, GCaMP7b/wrmScarlet ratio graphs are standardized to the control levels set to 1.0. (E) Confocal images of gonad arms from wild-type pGCS L4-stage larvae that were fed (top) or starved for 6 h (bottom), with GCaMP7b (green) and wrmScarlet (red) overlay. Note the punctate GCaMP7b signal in the distal side of the germ line for the 6 h starved L4 larva. Asterisks indicate the distal end of the gonad arm. Scale bar: 20 μm. Statistical analysis for A and B was with the Mann–Whitney test; for C was with the unpaired two-tailed Student's *t*-test; and for D was with Kruskal–Wallis with Dunn's multiple comparisons. For all figures, error bars show the mean±s.e.m. **P<0.01; ***P<0.001; ****P<0.0001. ns, not significant. Grayscale images for each channel in E are shown in Fig. S6.

Insulin signaling is known to promote germ cell proliferation in larvae (Michaelson et al., 2010). A null mutation of the insulin ligand *ins-3*(*ok2488*) significantly reduces larval germ cell proliferation (the mitotic index is ∼40% lower than in wild type) (Michaelson et al., 2010). We found that the level of Ca^2+^ in *ins-3*(*ok2488*); pGCS L4-stage mutants was reduced by 24.5% in the distal mitotic zone relative to wild-type levels (Fig. 4A). This reduction in Ca^2+^ level was present throughout the gonad (Fig. S4).

TGF-β signaling is also known to promote the mitotic proliferation of germ cells both through increasing GLP-1 expression in the DTC and through GLP-1-independent pathways (Dalfó et al., 2012; Pekar et al., 2017). *daf-1*(*m40ts*) is a temperature sensitive mutation of the TGF-β type I receptor. Switching *daf-1*(*m40ts*) L4 larvae from 16°C (the permissive temperature) to 25°C (the restrictive temperature) for one day reduces the number of cells in the germline mitotic zone of the resulting early adults by 38.5% (Dalfó et al., 2012). We repeated this temperature-shift protocol with *daf-1*(*m40ts*); pGCS mutants and found that the level of Ca^2+^ was reduced by 35.5% relative to wild-type levels (Fig. 4B).

The main mechanism by which TGF-β signaling promotes increased mitotic germ cell numbers in wild-type L4 larvae is by preventing entry into meiosis by promoting the expression of LAG-2 in the DTC (Pekar et al., 2017). To assess the role of TGF-β signaling on germ cell proliferation, we analyzed *daf-1*(*m40ts*) early larvae mutants, before germ cells enter meiosis in mid-L3 stage (Korta and Hubbard, 2010). Starvation-arrested L1 *daf-1*(*m40ts*) mutants with *ruIs32*[GFP::H2B] (Praitis et al., 2001) were placed on *Escherichia coli* OP50 plates at 25°C for 24 h and then the number of germ cells were counted. We observed ∼36% fewer germ cells in *daf-1*(*m40ts*); *ruIs32* compared to wild type; *ruIs32* (Fig. S5B). This was accompanied by a ∼21% reduction in Ca^2+^ levels in *daf-1*(*m40ts*) relative to wild type (Fig. S5A). Therefore, in the early larvae, inactivation of *daf-1* reduces germ cell proliferation and this is correlated with lower Ca^2+^ levels.

Loss of FOLR1-mediated folate signaling produces germ cells that are unable to increase their proliferation rate in response to folate obtained from ingested bacteria (Chaudhari et al., 2016). We found that *folr-1*(*ek44*) null mutant L4 larvae have a 13% reduction in basal Ca^2+^ levels (Fig. 4C). Thus, inactivation of three different signaling pathways that result in reduced germ cell proliferation rates were each correlated with reduced levels of basal cytoplasmic Ca^2+^.

Starvation significantly reduces germ cell proliferation in *C. elegans* (Angelo and Van Gilst, 2009; Seidel and Kimble, 2011, 2015). We tested the effect of starvation on germline Ca^2+^ levels. Early adults (∼1 day old with one row of 10 eggs or less) were starved for 3 h or 6 h. At 3 h, there was not a significant decrease in the germline basal Ca^2+^ levels (Fig. 4D). However, by 6 h, the basal Ca^2+^ levels were reduced by 33% relative to fed animals (Fig. 4D). When L4-stage animals were starved, they showed a more rapid reduction in basal cytoplasmic Ca^2+^ levels. The basal level of Ca^2+^ in 3 h starved L4 larvae was reduced by 38.5% relative to fed animals, and this reduced level was also observed at 6 h of starvation (a reduction of 35% relative to fed animals) (Fig. 4D). Thus, L4 larvae appear to have a more sensitive Ca^2+^ response to starvation than early adults, with L4 reaching a plateau of decreased basal cytoplasmic Ca^2+^ at 3 h of starvation that is similar to what early adults only reach at 6 h of starvation.

The intracellular localization of GCaMP7b signal in the germ cells of starved wild-type animals was different than in fed wild type or in the mutants with reduced germ cell proliferation described above. In addition to the reduction in overall GCaMP7b signal relative to wrmScarlet, the remaining GCaMP7b signal was enriched in puncta that localized to the cell periphery (in contrast to a more uniform signal throughout the cytoplasm) (Fig. 4E; Fig. S6). Overall, four different methods of reducing germ cell proliferation all led to decreases in basal cytoplasmic Ca^2+^ levels.

### Tumorous overproliferation is associated with increased basal Ca^2+^ levels

**Fig. 5.**
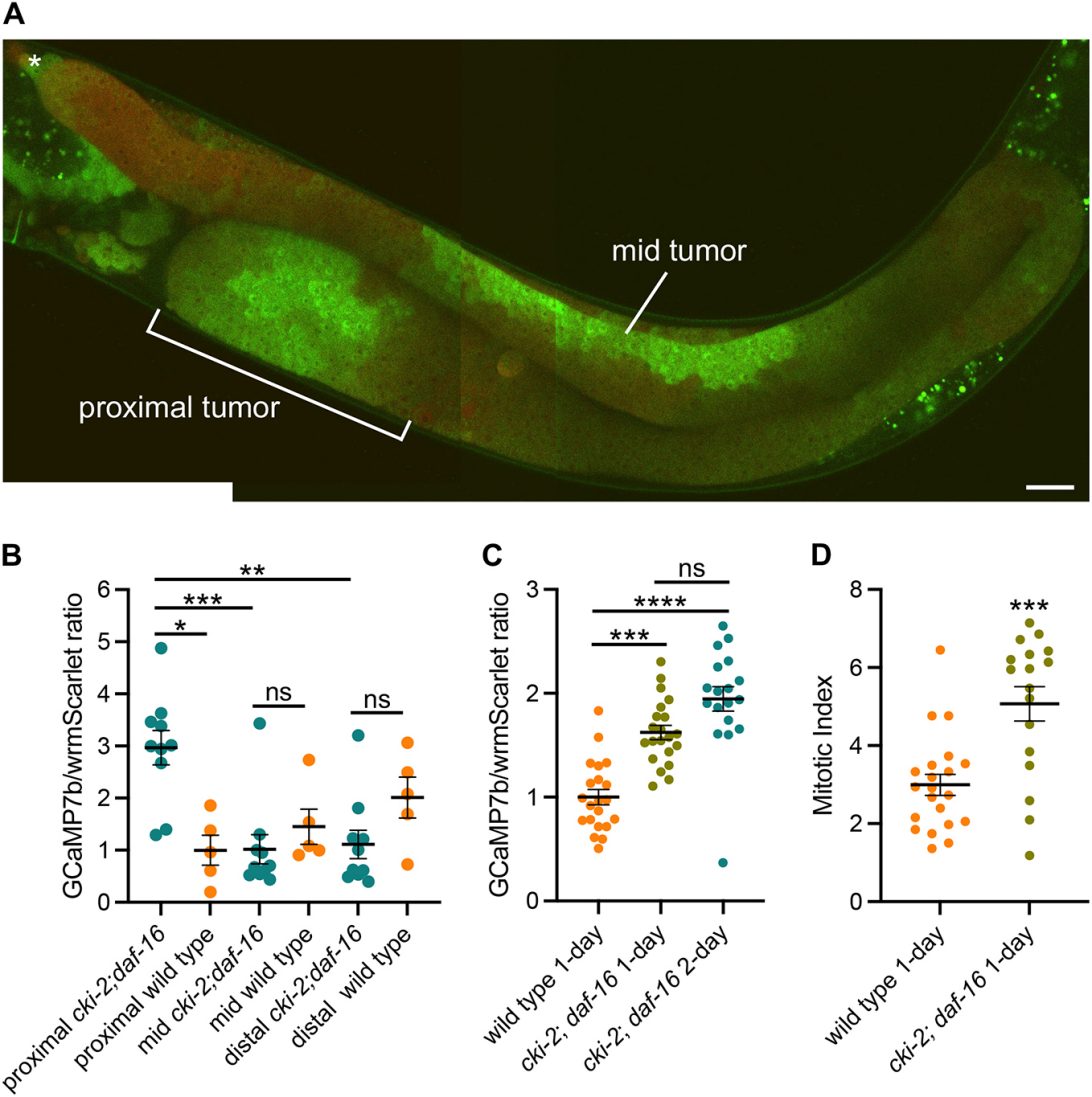
***cki-2*; *daf-16* mutants have increased Ca^2+^ in their proximal tumors.** (A) Composite confocal image of GCaMP7b (green) and wrmScarlet (red) overlay of tumors in the gonad arm of a *cki-2*; *daf-16*; pGCS adult hermaphrodite grown at 25°C for 48 h. Tumors are labeled. The distal tip of the gonad arm is marked with an asterisk. The focal plane varies slightly between the three individual images of the compound image. Scale bar: 20 μm. (B) Graph of GCaMP7b/wrmScarlet ratios in the proximal region or proximal tumor, mid region (without tumors) or distal mitotic zone of the germ line for *cki-2*; *daf-16*; pGCS and wild-type pGCS. (C) Graph of GCaMP7b/wrmScarlet ratios in the distal mitotic zone of 1-day-old adult wild-type pGCS and in the proximal tumors of 1-day-old and 2-day-old adult *cki-2*; *daf-16*; pGCS mutants at 25°C. (D) Graph of mitotic index in mitotic zone of 1-day-old adult wild-type pGCS, and in the proximal tumor of 1-day-old adult *cki-2*; *daf-16*; pGCS at 25°C. Statistical analysis for B and C was with the Kruskal–Wallis test with Dunn's multiple comparisons. Statistical analysis for D was with the Mann–Whitney test. *P<0.05; **P<0.01; ***P<0.001; ****P<0.0001. ns, not significant. Data are mean±s.e.m. Grayscale images for each channel in A are shown in Fig. S7.

To assess whether increased proliferation rates lead to an increase in Ca^2+^ levels in hermaphrodites, we analyzed proximal tumors in the *cki-2*(*ok2105*) and *daf-16*(*mu86*); pGCS strain. CKI-2 is a CDK inhibitor and DAF-16 is a FOXO transcription factor, both of which act to inhibit germ cell proliferation (Kalchhauser et al., 2011; Michaelson et al., 2010). Previously, we had utilized a *cki-2*(*ok2105*); *daf-16*(*mu86*); *glp-1*(*ar202ts*) triple mutant strain that produces large germline tumors when the animals are shifted from 16°C to 25°C (Chaudhari et al., 2016). We found that the *cki-2*(*ok2105*); *daf-16*(*mu86*); pGCS double mutant strain produced proximal germline tumors when shifted from 16°C to 25°C (Fig. 5A; Fig. S7). Animals were shifted for 2 days to 25°C, and the adults (all with visible tumors) were analyzed for the level of Ca^2+^. The proximal tumors in *cki-2*; *daf-16*; pGCS animals had significantly elevated levels of Ca^2+^ relative to the mid or distal regions of the gonad arms (Fig. 5B). The Ca^2+^ levels in proximal tumors were also significantly higher than in the proximal regions of wild-type pGCS animals (Fig. 5B).

We observed that 1-day-old *cki-2*; *daf-16* adults at 25°C had more uniform distributions of mitotic cells throughout the tumor region than larger 2-day-old tumors. The 1-day-old *cki-2*; *daf-16* proximal tumors had ∼69% higher mitotic index than the distal mitotic zone of 1-day-old wild-type adults, and ∼62% higher Ca^2+^ levels (Fig. 5C,D). Thus, the elevated Ca^2+^ levels in the 1-day-old adult proximal tumors are associated with increased mitotic proliferation relative to the wild-type adult mitotic zone.

### Reductions in basal Ca^2+^ levels reduce proliferation

So far, we have shown that conditions that decrease proliferation have lower basal Ca^2+^ levels and conditions with increased proliferation have higher basal Ca^2+^ levels. We wanted to determine if Ca^2+^ levels were instructive for proliferation by reducing the level of basal germ cell Ca^2+^ and determining the effect on proliferation. To reduce Ca^2+^ levels in the germ line, we inactivated the TRPM7-related Ca^2+^ channel GON-2.

**Fig. 6.**
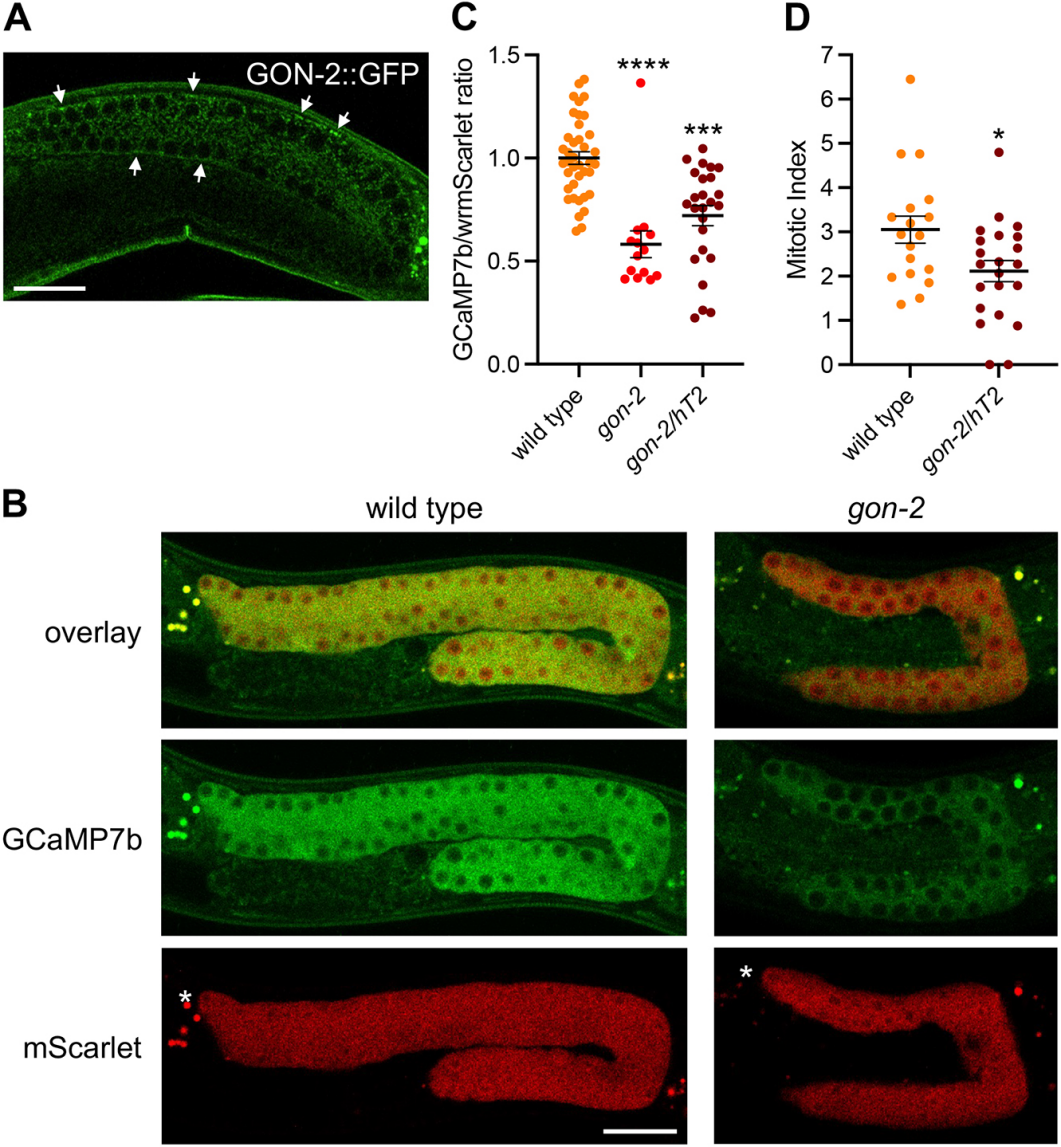
***gon-2* null mutants have reduced basal cytoplasmic Ca^2+^ levels and germ cell numbers.** (A) Confocal image showing expression of *gon-2p*::GON-2::GFP in the germ line of an adult hermaphrodite, with enrichment at the outer edge of the germ cells (arrows). (B) Confocal images of L4-stage wild-type and *gon-2*(*ok465*) gonad arms with GCaMP7b and wrmScarlet presented separately and overlaid. Asterisks indicate the distal end of the gonad arm. (C) Graph of the GCaMP7b/wrmScarlet ratio in *gon-2*(*ok465*) homozygotes and *gon-2*(*ok465*)/*hT2* heterozygotes. (D) Graph of the mitotic index of germ cells in the mitotic zone of *gon-2*(*ok465*)/*hT2* heterozygotes and wild type. Statistical analysis for C was with the Kruskal–Wallis test with Dunn's multiple comparisons. Statistical analysis for D was with unpaired two-tailed Student's *t*-test. *P<0.05; ***P<0.001; ****P<0.0001. Data are mean±s.e.m. Scale bars: 20 μm.

The *gon-2* gene encodes a TRPM-related Ca^2+^ channel, the closest human homolog of which is TRPM7 (West et al., 2001). *gon-2* mutants were first identified based on temperature-sensitive mutants in which the somatic gonad precursors do not divide at the restrictive temperature to form the normal hermaphrodite gonad structure (Sun and Lambie, 1997). A GON-2::GFP transgene (with GFP inserted in the genomic *gon-2* locus) demonstrates the expression of GON-2::GFP in germ cells, with an enrichment on the outer surface of the germ cells (Fig. 6A). Unlike the temperature-sensitive mutations, *gon-2*(*ok465*) null homozygotes form the normal somatic gonad structure. We observed that *gon-2*(*ok465*); pGCS animals had a 42% reduction in the GCaMP7b/wrmScarlet ratio in the distal region of the gonad (Fig. 6B,C).

Significantly, *gon-2*(*ok465*) mutants have a severe reduction in the number of germ cells relative to wild type and are sterile (Fig. 6B) (Peesapati et al., 2024). Late-stage L4 *gon-2*(*ok465*); pGCS homozygotes had only 30.9±2.8 (mean±s.e.m.) germ cells (*n*=14) on average in the region of the germ line that lies along the dorsal side. In contrast, wild-type late-stage L4 hermaphrodites had 184.6±12.6 germ cells (*n*=10) in the region of the germ line that lies along the dorsal side, which is an almost 6-fold greater number of germ cells than for late-stage L4 *gon-2*(*ok465*) mutants; *P*<0.0001.

Potentially, the decrease in Ca^2+^ levels in *gon-2*(*ok465*) mutants arose from having too few germ cells in a sterile germ line. To assess this, we determined the level of Ca^2+^ in *gon-2*/*hT2* heterozygotes, which are fertile and have relatively normal numbers of germ cells (124.2±8.1 germ cells per mitotic zone in L4 larvae, *n*=16). *gon-2*/*hT2* heterozygotes have a 28% lower level of Ca^2+^ in the distal mitotic zone relative to wild type (Fig. 6C). Therefore, *gon-2* heterozygotes with an overtly normal germ line also exhibit reduced germ cell basal Ca^2+^ levels, with a partial loss of GON-2 levels producing a partial reduction in basal cytoplasmic Ca^2+^ levels. The reduction in Ca^2+^ in *gon-2*/*hT2* heterozygote animals correlates with a 31% lower mitotic index than in wild type (Fig. 6D). Thus, the GON-2 Ca^2+^ channel is required to maintain basal cytoplasmic Ca^2+^ levels in germ cells, and reductions in Ca^2+^ levels when GON-2 is inactivated are associated with corresponding decreases in germ cell proliferation.

### Increases in basal Ca^2+^ levels increase proliferation

Having shown that decreases in basal cytoplasmic Ca^2+^ lead to reduced proliferation, we wanted to determine whether increases in Ca^2+^ levels would increase proliferation. To accomplish this, we focused on two essential regulators that remove cytoplasmic Ca^2+^: the SERCA channel, which transports cytoplasmic Ca^2+^ into the endoplasmic reticulum; and the Plasma Membrane Ca^2+^ ATPase (PMCA), which transports cytoplasmic Ca^2+^ out of the cell. There is only one SERCA gene in *C. elegans*: *sca-1* (Hughes et al., 2019). There are three PMCA genes in *C. elegans*: *mca-1*, *mca-2* and *mca-3* (Kraev et al., 1999). Of these three genes, only *mca-3* is expressed to high levels in the germ line (Ortiz et al., 2014). Partial inhibition of *sca-1* or *mca-3* are known to increase cytoplasmic Ca^2+^ levels in other *C. elegans* tissues (Hughes et al., 2019; Seidenthal et al., 2025).

**Fig. 7.**
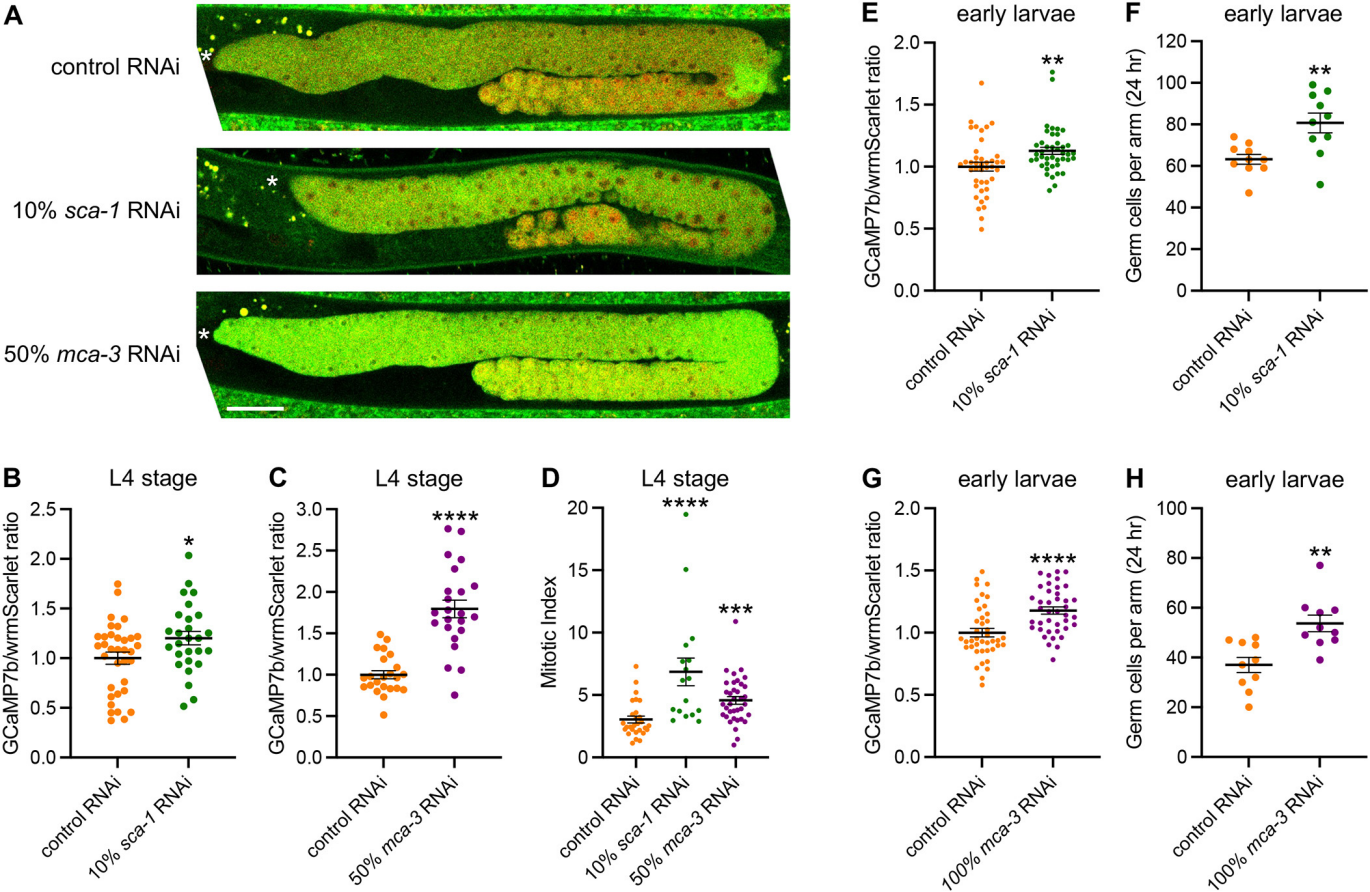
**Increasing basal cytoplasmic Ca^2+^ increases germ cell proliferation.** (A) Confocal images of control, 10% *sca-1* and 50% *mca-3* RNAi gonad arms in pGCS L4 larvae with overlaid GCaMP7b (green) and wrmScarlet (red). Sections of the images are cut off at the edge because the images were angled before cropping. Asterisks indicate the distal end of the gonad arm. Scale bar: 20 μm. (B,C) Graph of the GCaMP7b/wrmScarlet ratios in pGCS L4 larvae subject to 10% *sca-1* or control RNAi (B) or 50% *mca-3* or control RNAi (C). (D) The mitotic index of germ cells in the mitotic zone of pGCS L4 larvae treated with control RNAi, 10% *sca-1* RNAi or 50% *mca-3* RNAi. (E,G) Graphs of the GCaMP7b/wrmScarlet ratios in a germline-specific RNAi strain with pGCS that was treated with control RNAi or with 10% *sca-1* (E) or 100% *mca-3* (G) and grown for 24 h. (F,H) Corresponding graphs of the number of germ cells per gonad arm in germline-RNAi-specific larvae grown under the same conditions as E and G, respectively. In each gonad arm, germ cells arise from a single germ cell precursor cell. Statistical analysis for B, C, F, G and H was with unpaired two-tailed Student's *t*-test. Statistical analysis for D was with the Kruskal–Wallis test with Dunn's multiple comparisons. Statistical analysis for E was with the Mann–Whitney test. *P<0.05; **P<0.01; ***P<0.001; ****P<0.0001. Data are mean±s.e.m. Grayscale images for each channel in A are shown in Fig. S8.

We observed that 10% *sca-1* RNAi (i.e. 10% *sca-1* RNAi bacteria mixed with 90% control RNAi bacteria for feeding RNAi) increased basal cytoplasmic Ca^2+^ levels in distal germ cells by 20% relative to control RNAi in L4-stage larvae (Fig. 7A,B; Fig. S8), and 50% *mca-3* RNAi increased basal cytoplasmic Ca^2+^ levels in distal germ cells by 79% relative to control RNAi in L4 larvae (Fig. 7A,C; Fig. S8).

We next asked whether these increases in basal cytoplasmic Ca^2+^ levels were associated with increased proliferation in L4-stage larvae. Notably, both 10% *sca-1* RNAi and 50% *mca-3* RNAi significantly increased the mitotic index of germ cells in the mitotic zone relative to wild type (Fig. 7D). To more directly assess proliferation, we wanted to determine how many germ cells were produced during the 24 h period (at 20°C) after starvation-arrested L1 larvae were placed on food. However, we observed that 10% *sca-1* and 50% or 100% *mca-3* RNAi significantly slowed larval development, which indirectly affected the rate of germ cell proliferation. To bypass this issue, we performed the RNAi knockdown only in the germline, using a germline-specific RNAi strain in which an *rde-1* mutant globally inactivates RNAi but is combined with germline expression of RDE-1 to restore RNAi in the germline (Zou et al., 2019). With this approach, we observed more cells generated during the 24 h period for 10% *sca-1* RNAi (∼28% more cells) and for 100% *mca-3* RNAi (∼45% more cells) (Fig. 7E-H). Thus, we conclude that increasing basal cytoplasmic Ca^2+^ levels through two different cellular mechanisms increases germ cell proliferation.

## DISCUSSION

Here, we provide the first report of the levels of cytoplasmic Ca^2+^ throughout the *C. elegans* germ line using a stably integrated ratiometric Ca^2+^ reporter. The use of an integrated ratiometric Ca^2+^ reporter allows more accurate comparisons of the level of Ca^2+^ during development. Our analysis shows that there are changes in Ca^2+^ levels during sexual development, with a reduction in the Ca^2+^ levels of spermatogenic cells and an increase in oocytes.

We observe a striking increase in Ca^2+^ levels in a subset of cells in the bend region of mid-to-late L4 larvae. The distribution of elevated-Ca^2+^ cells in the L4.4-L4.9 sub-stages (Fig. S3) suggests that the elevated Ca^2+^ state is transient within the ∼4.7 h L4.4-L4.9 developmental window at 20°C (Mok et al., 2015), potentially peaking at ∼L4.7, the only sub-stage in which all gonad arms examined (8/8) contained elevated-Ca^2+^ cells.

Fully feminizing the germline, through mutations in fem or fog genes, eliminates the elevated-Ca^2+^ cells. Conversely, masculinization of the germline, with mog mutants, reduces the number of elevated-Ca^2+^ cells and the percentage of gonads with elevated-Ca^2+^ cells. The elevated-Ca^2+^ cells are located between proximal cells undergoing spermatogenesis and distal cells initiating the oogenic program (Ellis and Schedl, 2007) and, as described above, both programs are required for their full appearance. These findings suggest a model in which some germ cells at the interface between the spermatogenic and oogenic programs elevate Ca^2+^ levels in response to the spermatogenesis-to-oogenesis transition and/or to local interactions between these programs.

There is an increase in the level of Ca^2+^ in the distal mitotic zone during late L4 and the young adult stage. Thus, normal mitotic proliferation is associated with increased Ca^2*+*^ levels. Moreover, we have observed that conditions that reduce the proliferation rates of germ cells correlate with reductions in basal cytoplasmic Ca^2+^ levels. These conditions include loss of insulin, TGF-β and FOLR1 signaling, and starvation. Conversely, germline tumors, which have increased proliferation, show elevated basal cytoplasmic Ca^2+^ levels. These correlations suggest either that basal Ca^2+^ levels are responsive to the proliferation rate (with Ca^2+^ levels increasing or decreasing in response to increased or decreased proliferation rates, respectively) and/or that Ca^2+^ levels are regulated by signaling events that also alter proliferation rates (potentially by altering Ca^2+^ levels). These are not mutually-exclusive scenarios, and both could be operating in different physiological contexts.

Our data on starvation suggests that reductions in Ca^2+^ levels do not directly follow from a reduction in the rate of proliferation, but may instead respond to other cues, such as metabolism. In early adult hermaphrodites, basal Ca^2+^ levels did not change at 3 h of starvation but were significantly reduced by 6 h. In contrast, L4 larvae showed a significant reduction in basal Ca^2+^ at 3 h that was similar to 6 h, suggesting that Ca^2+^ levels had already reached a reduced plateau by 3 h. These results do not correlate with published observations on the reduction in M-phase cell numbers. In starved early adults, the number of M-phase cells per mitotic zone drops by about half within the first hour and is near zero by 3 h, when we observe no effect on basal Ca^2+^ levels (Seidel and Kimble, 2015). In contrast, in starved L4 larvae, M-phase cell numbers are reduced by less than 50% at 3 h, when we observe a significant drop in Ca^2+^ levels (Seidel and Kimble, 2015). Therefore, the differential reductions in basal Ca^2+^ levels between the two developmental stages do not correlate with the relative changes in the number of M-phase cells per mitotic zone. A plausible hypothesis is that the decrease in cytoplasmic Ca^2+^ levels during starvation is in response to reduced metabolism or metabolism-associated signaling. Notably, adults have significantly more fat storage than L4 larvae, with the triacylglyceride-to-protein ratio twice as high in early adults compared to L4 larvae (Palgunow et al., 2012). Thus, early adults have substantially more fat stores that are likely to delay the effects of starvation on metabolism relative to L4 larvae. Additionally, because germ cells divide faster in L4 larvae than in adults (Roy et al., 2016), the earlier response of Ca^2+^ levels to starvation in L4 larvae could reflect a greater sensitivity of the rapidly proliferating larval germline to starvation-induced changes in metabolism or nutrient-responsive signaling.

We observed that decreasing Ca^2+^ levels, by inactivating the GON-2 Ca^2+^ channel, decreases proliferation. Conversely, increasing Ca^2+^ levels, by partially inactivating the SERCA and PMCA mechanisms for removing cytoplasmic Ca^2+^, led to increases in germ cell proliferation. This suggests that the level of basal cytoplasmic Ca^2+^ is instructive for the rate of germ cell proliferation. This data supports a model in which the level of basal cytoplasmic Ca^2+^ acts as a rheostat to modulate the rate of germ cell proliferation.

Our study suggests that the TRPM7-channel GON-2, which is expressed in the germ line, is a central regulator of basal cytoplasmic Ca^2+^ levels. Inactivation of *gon-2* leads to a 42% reduction in the level of basal cytoplasmic Ca^2+^ in the distal region of the gonad. This significant reduction in cytoplasmic Ca^2+^ levels is associated with very few germ cells and sterility. That the basal level of Ca^2+^ in germ cells only goes down 42% suggests that other Ca^2+^ channels/transporters also function to transport Ca^2+^ into the germ line, and that these other channels/transporters are unable to compensate for a complete lack of GON-2 to bring cytoplasmic Ca^2+^ to normal physiological levels.

It is known that transient increases in Ca^2+^ that are associated with cell signaling can promote cell cycle progression in mammalian cells (Humeau et al., 2018). Transient changes in Ca^2+^ linked to signaling are also associated with proliferation in the invertebrates *Drosophila melanogaster* and Planaria (Brodskiy et al., 2019; Martelly et al., 1983). While there are hundreds of studies on transient Ca^2+^ increases in response to signaling leading to increased cell proliferation, there are relatively few studies that demonstrate a correlation between proliferation and basal Ca^2+^ levels. Cultured primary human or rat pulmonary artery smooth muscle cells in culture have ∼2-fold higher basal cytoplasmic Ca^2+^ level when proliferating in the presence of serum than when quiescent in serum-deprived conditions (Golovina, 1999; Platoshyn et al., 2000). Additionally, raising the level of basal cytoplasmic Ca^2+^ by inhibiting store-operated Ca^2+^ entry in a mouse myoblast cell line increases proliferation (Kim et al., 2019). While these few examples link basal cytoplasmic Ca^2+^ levels to proliferation in mammalian cells, our study shows that basal Ca^2+^ levels regulate proliferation in a tissue in an intact animal under physiological conditions. Here, we have found that normal physiological contexts of food intake and cell signaling pathways regulate the basal level of cytoplasmic Ca^2+^ to affect the rate of germ stem cell proliferation. There are no other published studies linking Ca^2+^ levels (basal or transient) and cell proliferation in *C. elegans*.

Our study has thus identified that the basal cytoplasmic level of Ca^2+^ regulates cell proliferation in germ cells, and that changes in basal Ca^2+^ levels are associated with multiple pathways that regulate germ cell proliferation. It will be interesting in future experiments to determine how different signaling pathways intersect with or regulate basal cytoplasmic Ca^2+^ levels to control the proliferation of *C. elegans* germ stem cells.

## MATERIALS AND METHODS

### *C. elegans* strains and culture methods

*C. elegans* strains were cultured according to established methods (Sulston and Hodgkin, 1988). Strains used in this study are listed in Table S1.

The GCaMP7b signal from pGCS is not readily visible with a stereomicroscope equipped with fluorescence, but is easily observed with a confocal microscope, where *z*-sections can separate the green germline signal from the green autofluorescence of the intestine. Strains with the pGCS transgene spontaneously lose expression over time, potentially due to negative selective pressure from expression of GCaMP7b. To counteract this, actively growing reporter strains were maintained by weekly selection for animals with high levels of wrmScarlet expression. Strains that have lost high-level reporter expression can have their expression restored by sequentially selecting higher levels of expression in subsequent generations.

For selection of animals with higher pGCS expression, ∼12 animals are placed on microscope slides with agar pads in 6 μl of M9 buffer (Sulston and Hodgkin, 1988). The level of wrmScarlet is assessed with a compound fluorescence microscope. Animals with higher wrmScarlet expression are recovered by sliding the coverslip and transferring the worms with a curved platinum-iridium wire worm pick to a nematode growth medium (NGM) agar plate with bacteria (Sulston and Hodgkin, 1988).

Starvation was carried out by washing animals of the selected developmental stage six times in M9 buffer using polystyrene 15 ml tubes (Falcon). The animals were then placed on M9 agarose plates, containing M9 buffer with 15 g/ml of agarose (Sigma-Aldrich), and starved for the specified time.

To obtain proximal tumors in *cki-2(ok2105)*; *daf-16(mu86)*; pGCS strains; animals were shifted from 16°C to 25°C for 1 or 2 days. Animals with obvious tumors (encompassing the majority of adults) were selected for analysis.

### RNAi

RNAi was carried out with feeding-RNAi bacterial strains from the Ahringer library (Kamath et al., 2003). RNAi bacteria were grown (from an overnight culture) in 2× yeast extract tryptone (2×YT) media plus 100 μg/ml carbenicillin to an optical density at wavelength 600 nm (OD_600_) of between 0.4 and 0.6, at which time 1 mM IPTG (Gold Biotechnology) was added, and the culture was grown for 6 h. The bacteria were concentrated by centrifugation at 3724 ***g*** (4°C) and resuspension in a smaller volume of lysogeny broth (LB) media with 100 μg/ml carbenicillin and 1 mM IPTG, and plated onto NGM agar plates with 100 μg/ml carbenicillin and 1 mM IPTG. For RNAi with reduced effectiveness, the bacteria with the RNAi of interest were mixed with control RNAi after centrifugation (with the relative amounts normalized based on OD_600_ measurements) before plating on NGM agar plates. So, for example, 10% *sca-1* RNAi involved mixing 1 part *sca-1* RNAi feeding bacteria with 9 parts of control (pPD129.36 vector) RNAi feeding bacteria. For most experiments, L4-stage hermaphrodites were placed on RNAi and their progeny were analyzed.

Both *sca-1* and *mca-3* complete loss of function produce embryonic and early larval arrest as reported in WormBase (Sternberg et al., 2024). The 10% *sca-1* RNAi condition was based on a publication that showed that 10% *sca-1* RNAi extended lifespan (García-Casas et al., 2021). The 50% *mca-3* RNAi condition was the highest *mca-3* RNAi concentration tested that did not overtly affect development.

### Generation of the *pie-1p*::GCaMP7b::P2A::wrmScarlet integrated reporter

The GCaMP7b sequence with three PATC introns and 120 bp flanking sequences, for insertion into the *pie-1p*::*dpy-10* crRNA SKI LODGE locus in strain WBM1119, was synthesized by BioMatik Inc. Recombinant HIS6-Cas9 protein was biochemically isolated by our lab as previously described (Peesapati et al., 2024), using plasmid pHO4d-Cas9 (Fu et al., 2014). The *dpy-10* crRNA, sequence GCUACCAUAGGCACCACGAG, was previously described (Silva-Garcia et al., 2019) and was synthesized by Synthego. CRISPR/Cas9 was assembled *in vitro* according to Synthego instructions with 2.3 μM Cas9 and 2.4 μM annealed crRNA and tracrRNA (Synthego). The rescue DNA construct was an ssDNA-dsDNA-ssDNA annealing of the GCaMP7b sequence with the full GCaMP7b plus 120 bp flanking sequences so that the 120 nt flanking sequences were ssDNA and the GCaMP7b sequence was dsDNA. The annealing procedure for the ss-ds-ss DNA rescue construct was as described (Dokshin et al., 2018). The injection mix included 2.3 μM Cas9 and 2.4 μM annealed crRNA and tracrRNA and 118 ng/μl rescue DNA. Gravid 1-day old wild-type hermaphrodites were injected in their gonads with the CRISPR/Cas9 rescue mix. Dpy progeny (due to CRISPR/Cas9 inactivation of the endogenous *dpy-10* locus) were isolated and screened for the insertion using PCR. The *dpy-10* mutation was eliminated by outcrossing the strain.

The insertion of the P2A::wrmScarlet sequence into the *pie-1p*::GCaMP7b SKI LODGE site was undertaken using the same procedure. The CRISPR crRNA sequence at the 3′ end of the inserted GCaMP7b coding sequence was GGGCGCGAGATGTTACTTGG, and was synthesized by Synthego. The P2A::wrmScarlet DNA sequence was synthesized by Azenta. An ss-ds-ss DNA rescue construct for P2A::wrmScarlet was generated and included in the injection mix as described above. Dpy progeny were isolated and screened for insertions by PCR. The strain with the P2A::wrmScarlet insertion was outcrossed several times.

### Immunofluorescence

Mitotic cells in the gonad were identified by immunofluorescence using anti-phosphohistone H3 (Ser10) antibody (Invitrogen, MA5-15220; clone K.872.3; lot 79533006; RRID: AB_11008586). Fluorescence slides with 10 mm circles (Thermo Fisher Scientific, 22339408) were coated with 25 μl of 1 mg/ml poly-L-lysine (≥300,000 MW, P1524, Sigma-Aldrich) and then heated at 75°C for ≥15 min on a heat block. Animals were placed in 10 μl of M9 buffer with 0.2 mg/ml tetramisole (Sigma-Aldrich) within the 10 mm circle of a coated slide. Gonads were dissected by cutting animals behind the pharynx with a #15 scalpel blade (Bard-Parker). A 24×60 mm No. 1 coverslip (VWR, 48404454) was placed on the slide, and it was frozen on a heat block on dry ice. The coverslip was rapidly removed, and the dissected gonads were incubated with 3% formaldehyde (Sigma-Aldrich, 252549) in phosphate buffered saline (PBS) for 30 min at room temperature. Slides were washed five times with PBST [PBS plus 0.2% Tween-20 detergent (EMD Chemicals)] and once with PBS. Samples were blocked in 1% bovine serum albumin (BSA) in PBS for 1 h at room temperature and incubated at 4°C overnight in 1% BSA with 1:400 dilution of mouse monoclonal anti-phosphohistone H3 (Ser10) antibody (Invitrogen, MA5-15220; clone K.872.3; lot 79533006). Slides were washed five times with PBST and once with PBS, blocked for 15 min with 1% BSA in PBS, and incubated for 2 h at room temperature with 1:500 anti-mouse IgG1 nanobody Alexa Fluor 568 (ChromoTek/ProteinTech, sms1AF568-1; lot 920918046AF3; RRID: AB_2827579) in 1% BSA in PBS. Slides were washed five times with PBST and incubated for 15 min in 1 mg/ml p-phenylenediamine (PPD) anti-fade agent (Sigma-Aldrich) and 1 μg/ml Hoechst 33342 DNA stain (Sigma-Aldrich) in PBS, then mounted with 90% glycerol with 1 mg/ml p-phenylenediamine and 1 μg/ml Hoechst 33342.

### Microscopy and image analysis

Non-confocal fluorescence, immunofluorescence and differential interference contrast (DIC) images were obtained using a Zeiss Axioskop microscope, with images captured with either a Tucsen Dhyana 400 BSI V2 sCMOS camera using Micromanager software (v.1.4.23) or with a Hamamatsu ORCA-ER CCD camera using OpenLab 5.02. software (Improvision).

Confocal images for the analysis of GCaMP7b-wrmScarlet were taken on a Zeiss LSM 880 confocal microscope, captured with Zen Black software (Zeiss). The image for GON-2::GFP expression in the germline was obtained using an Andor Dragonfly Spinning Disk Confocal Microscope. Images were processed using Adobe Photoshop (v. 22.0.0) and Fiji software (ImageJ, v. 2.1.0/1.53s). Matched fluorescence images to be used for level comparisons were processed identically and did not include gamma adjustments. For GCaMP7b/wrmScarlet ratios, control wild-type animals were always imaged at the same session, and the control GCaMP7b/wrmScarlet ratios were used to standardize the experimental ratios. The distal mitotic zone region that was analyzed was as previously described for the different developmental stages (Pepper et al., 2003).

For the calculation of GCaMP7b/wrmScarlet ratios, mean background signals for each channel were obtained from an intra-animal region within the image that had low level of both GCaMP7b and wrmScarlet intensity. The mean background signals were subtracted from the mean GCaMP7b and wrmScarlet signals, and the signal-minus-background levels were used to calculate the GCaMP7b/wrmScarlet ratio.

Continuous distal-to-proximal GCaMP7b/wrmScarlet ratio profiles were generated in Fiji using the freehand line tool with a line width of 10. The line was drawn from the distal end to the proximal end of the germ line, and values along the line were obtained using the Plot Profile function of Fiji; each profile contained more than 400 values. Values along each line-scan profile were resampled to 201 equally spaced positions by linear interpolation. For presentation in Fig. 3, average line values were normalized such that the mean value in the 40-60% length interval from the distal end was set to 1. Statistical comparisons for different regions of the distal-to-proximal average line were performed by comparing the average values of each animal in the dataset for that decile to the average values of each animal in the dataset for the 40-60% interval using one-way ANOVA with Dunnett's multiple-comparisons test.

For the analysis of larvae grown for 24 h from L1-arrest, eggs from animals grown on 10% *sca-1* or 100% *mca-3* RNAi plates were isolated by alkaline hypochlorite treatment and then allowed to hatch in M9 buffer with 5 μg/ml cholesterol to obtain arrested L1 larvae (Sulston and Hodgkin, 1988). The arrested L1 larvae were placed on RNAi plates (for 10% *sca-1* RNAi) or on OP50 plates (for 100% *mca-3* RNAi). After 24 h at 20°C, L1 larvae were collected, washed twice with water and, for animals with the germline-specific RNAi background [*rde-1(mkc36)*; *mkcSi13*], the larvae were frozen in a drop of water on a slide at −80°C, lyophilized, permeabilized and fixed by adding drops of acetone and letting it air dry (twice), and then stained with 1 μg/ml Hoechst with 1 mg/ml PPD. *Z*-section images (1 μm spacing) were obtained on a Zeiss Axioskop microscope, and germ cells were counted based on their stereotypical genomic DNA morphology using the Cell Counter tool in Fiji. Alternatively, for animals with the germline-specific RNAi background and expressing PGL-1::GFP [*pgl-1(ax3122)*] (Putnam et al., 2019), the animals were resuspended in 15 mM levamisole/tetramisole to paralyze the larvae, mounted on a slide with an agar pad, and germ cells were counted based on the PGL-1::GFP fluorescent signal. Similar results were obtained with both approaches; the data shown in Fig. 7F,H were with Hoechst staining. For the analysis of *daf-1*(*m40ts*) germ cell counts in the early larvae, the same approach was used, except the genetic background included *ruIs32*[*pie-1p*::GFP::histoneH2B], and germ cells were counted based on GFP::histone H2B fluorescent signal.

For figure images that are to be quantitatively compared, the level of wrmScarlet signal was used to standardize the intensity of GCaMP7b and wrmScarlet signals to account for differences in expression level of the pGCS transgene. To accomplish this, the mean level of wrmScarlet signal for the entire distal-side of the germ line was determined. The ratio was calculated for the mean wrmScarlet level in the higher-expression image divided by the mean wrmScarlet level in the lower-expression image. The signal of both wrmScarlet and GCaMP7b channels in lower-expression images were raised by the ratio value using the Fiji/ImageJ multiply function. Once the level of expression was equalized between images, the images were leveled to the same extent.

The mitotic index was determined from immunofluorescence images of anti-phosphohistone H3 immunofluorescence co-stained with Hoechst 33342 DNA stain. Z-stacks of the distal end of the germline were acquired at 1-μm z-step intervals using a 63× objective on a Zeiss Axioskop microscope. The mitotic zone was defined as the region distal to the first row containing multiple nuclei with crescent-shaped Hoechst-stained DNA, which marks the leptotene/zygotene stage of entry into meiotic prophase (Hansen and Schedl, 2013). Nuclei were counted using the Cell Counter tool in Fiji. The mitotic index was calculated as the number of nuclei that were anti-phosphohistone H3-positive divided by the total number of nuclei in the mitotic zone.

### Statistics

Every experimental result was repeated with comparable results in at least two biological replicates that were carried out on different days. Test of normality was performed using the Shapiro–Wilk test in Prism 11 (v. 11.0.0). Statistical analyses between two parametric groups of quantitative measurements were performed using unpaired two-tailed Student's *t*-tests in Excel (v.16.108). Statistical analyses between two non-parametric groups of quantitative measurements were performed using the Mann–Whitney test in Prism 11. Statistical analyses for comparisons between two groups of proportions were performed with Fisher's exact test in Prism 11. Statistical comparisons among multiple parametric groups were performed using one-way ANOVA with Dunnett's multiple-comparisons in Prism 11. Statistical comparisons among multiple non-parametric groups were performed using the Kruskal–Wallis test with Dunn's multiple comparisons in Prism 11. Results are reported as mean±s.e.m. (standard error of the mean). For all figures, *P<0.05; **P<0.01; ***P<0.001; ****P<0.0001.

## Supplementary Material



10.1242/develop.205301_sup1 Supplementary information
