# Supplementary material for: Basal cytoplasmic calcium levels regulate C. elegans germ stem cell proliferation
Source: Development. 2026 Jul 8;153(13):dev205301. doi: 10.1242/dev.205301 (PMC13380967; doi:10.1242/dev.205301)
Supplement: Supplementary information [file develop-153-205301-s1.pdf]

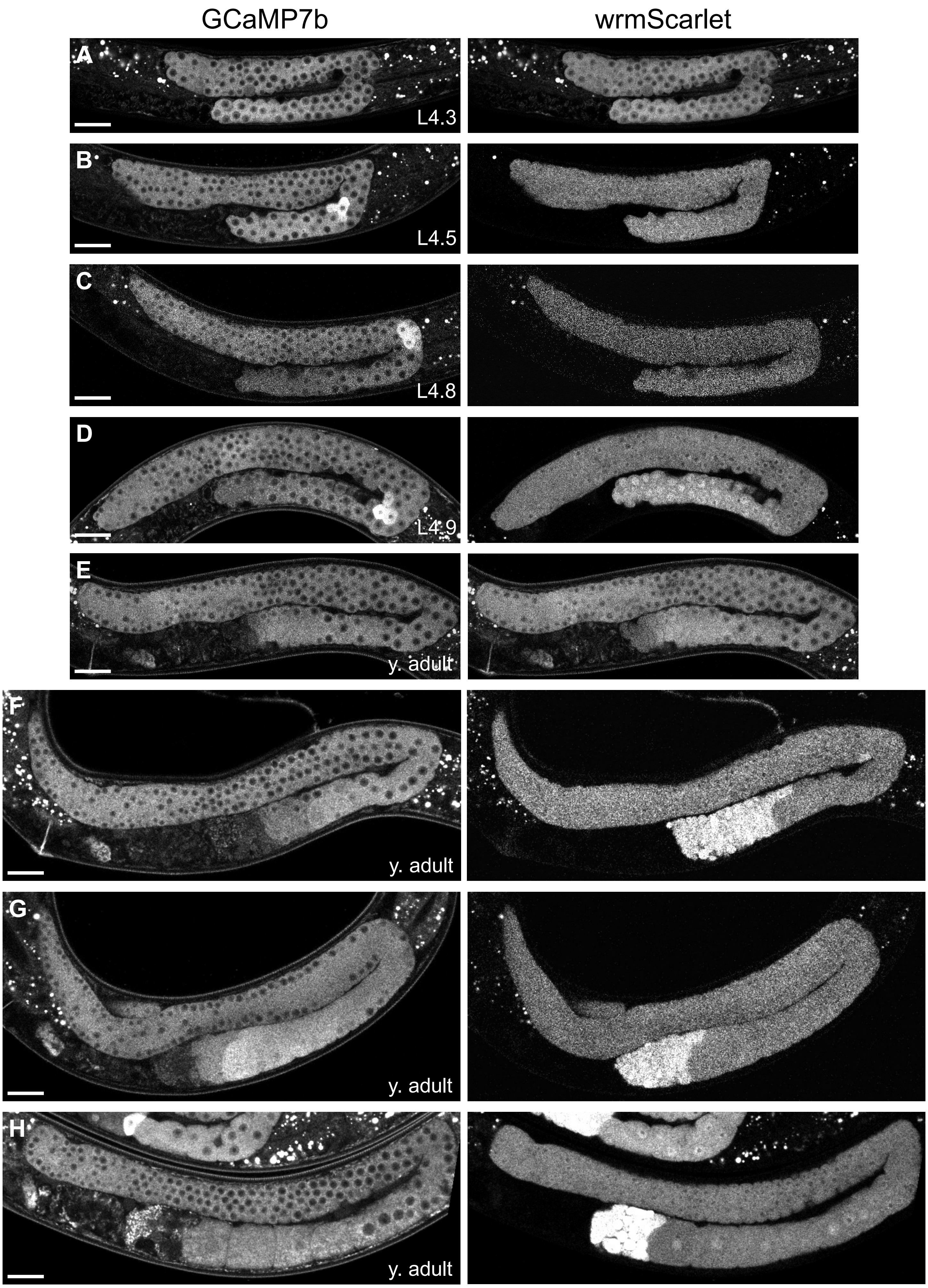

Fig. S1. Single-channel grayscale images of GCaMP7b and wrmScarlet for the overlaid images in Figure 2. Scale bars, 20 μm.

Development • Supplementary information

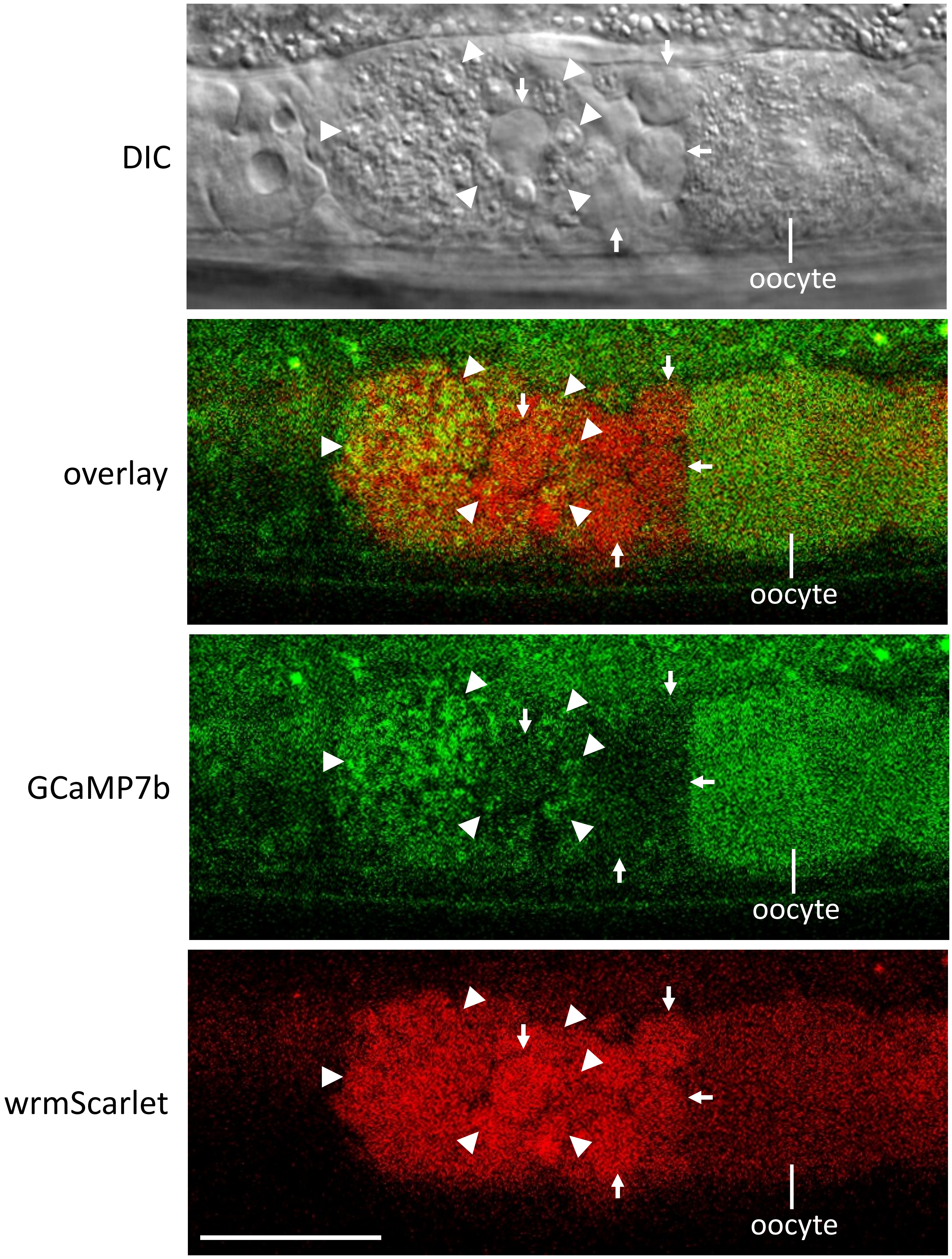

Fig. S2. Sperm and proximal oocyte of wild-type pGCS young adult hermaphrodite. DIC image and confocal images of GCaMP7b (green), wrmScarlet (red), and overlay. Arrowheads, sperm. Arrows, residual bodies. Scale bar, 20 µm.

Development • Supplementary information

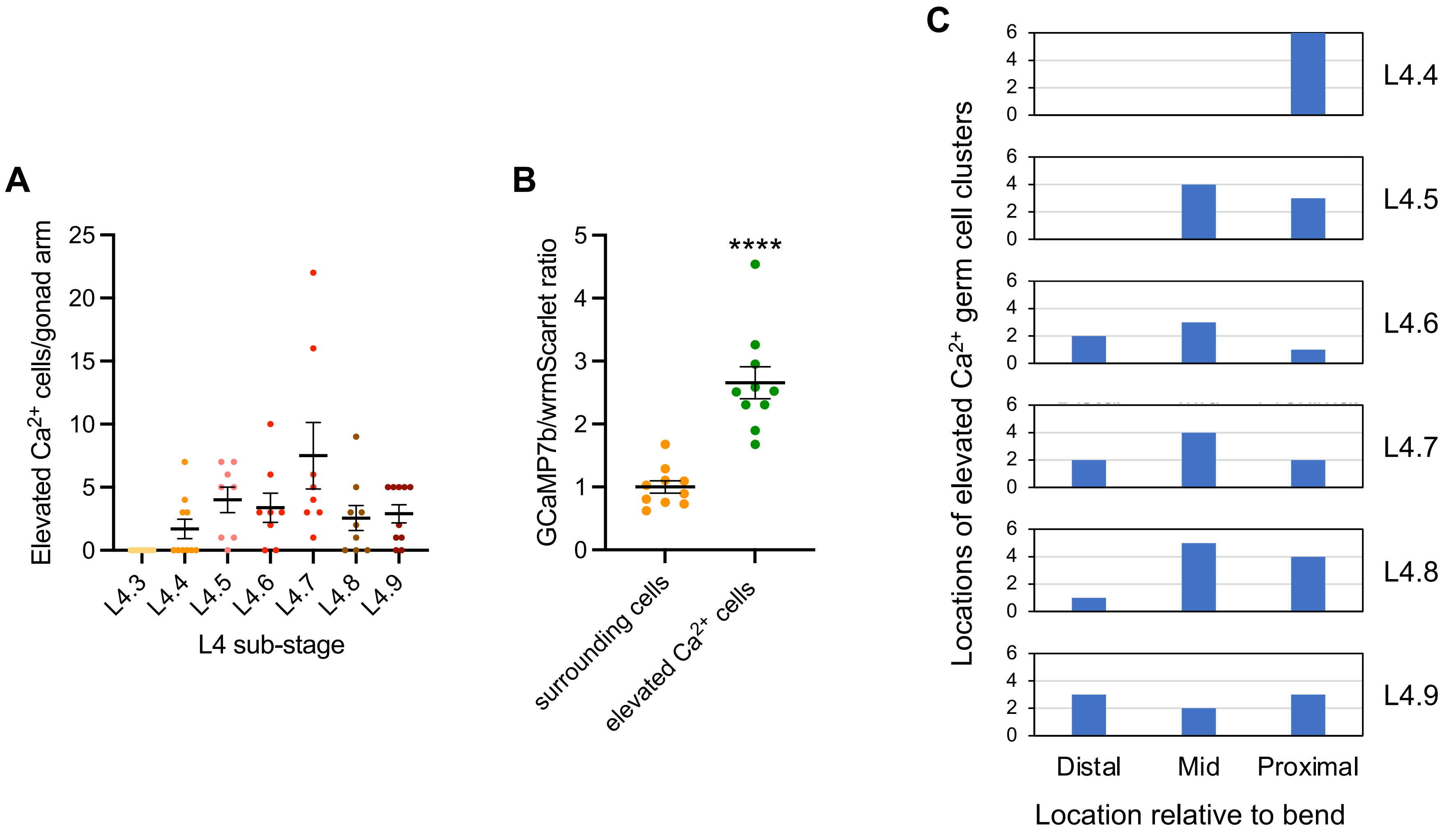

Fig. S3. Analysis of cells with elevated Ca^{2+} in L4-stage germlines. **A)** Graph of the number of elevated-Ca^{2+} germ cells observed in L4.3 to L4.9 sub-stages. The number of gonad arms with elevated-Ca^{2+} cells that were analyzed for the seven stages, respectively, were n = 8; 10; 8; 8; 8; 9; and 10. **B)** Graph of the GCaMP7b/wrmScarlet for elevated-Ca^{2+} germ cells and surrounding germ cells. Statistical analysis was with unpaired two-tailed Student's t-test. **C)** Graph of the localization of elevated-Ca^{2+} germ cells in L4.4 to L4.9 sub-stages. Locations are distal to, proximal to, or touching (Mid) a line drawn through the middle of the bend of the gonad along the anterior-posterior axis.

Development • Supplementary information

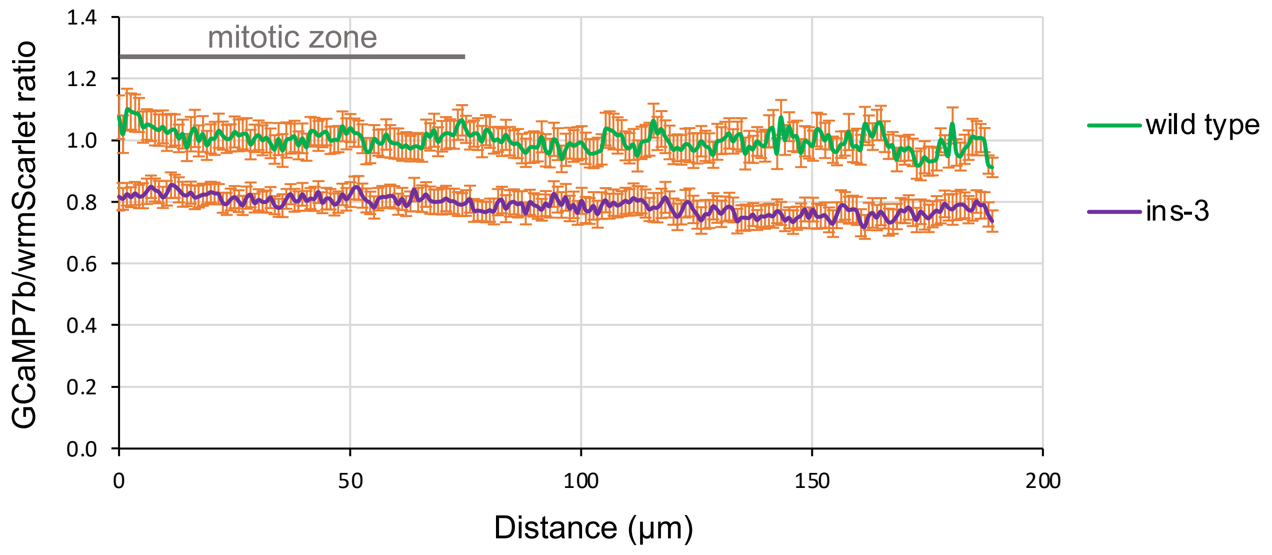

Fig. S4. A line graph of the distal-to-proximal Ca^{2+} levels along the distal gonad arms of *ins-3*(*ok2488*); pGCS and wild-type pGCS L4 larvae. The approximate length of the distal mitotic zone in these larvae is marked. The number of gonad arms analyzed were n = 23 for *ins-3*(*ok2488*); pGCS, and n = 24 for wild-type pGCS. The average values for distal-side gonad lines differed significantly between the two genotypes ($p < 0.0001$, Student's t-test).

Development • Supplementary information

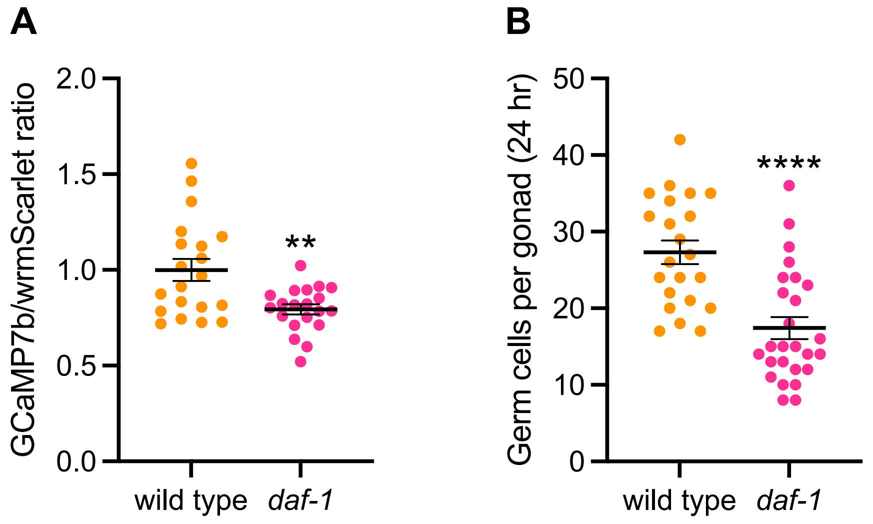

Fig. S5. Germ cell proliferation and Ca^{2+} levels in *daf-1* mutant early larvae. **A)** Graph of the GCaMP7b/ wrmScarlet ratios in wild-type pGCS and *daf-1*(*m40ts*); pGCS larvae that were starvation arrested and then placed on OP50 bacteria for 24 hrs at 25°C before imaging. **B)** Graph of the number of germ cells per gonad in wild-type and *daf-1*(*m40ts*) larvae carrying *ruIs32*[GFP::H2B] that were starvation arrested and then placed on OP50 bacteria for 24 hrs at 25°C before analysis. The *ruIs32* transgene slows animal development; therefore, the animals were at the L2 larval stage when analyzed. Statistical analysis for (**A**) was with unpaired two-tailed Student's t-test. Statistical analysis for (**B**) was with the Mann–Whitney test.

Development • Supplementary information

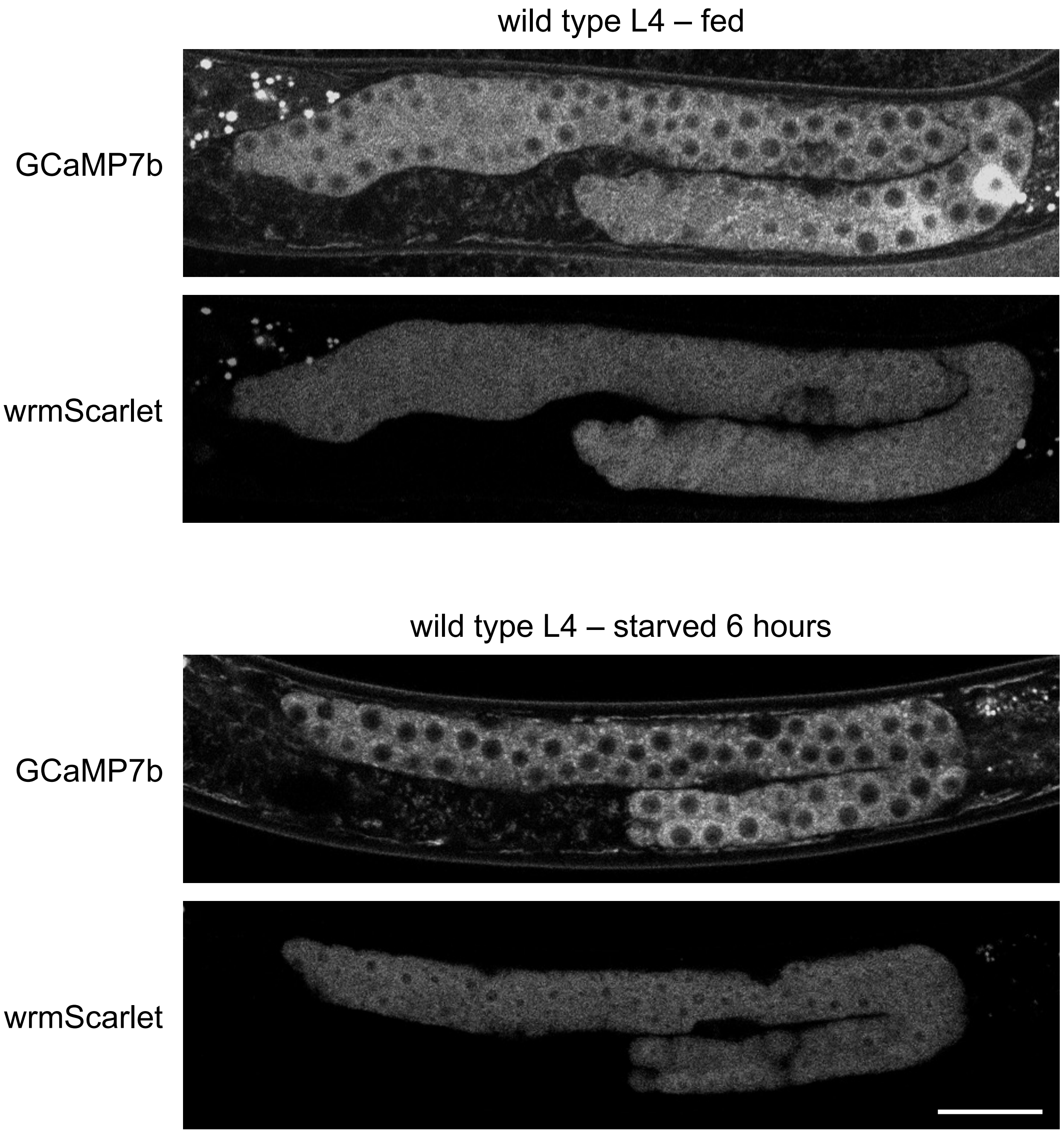

Fig. S6. Single-channel grayscale images of GCaMP7b and wrmScarlet for the overlaid images in Figure 4E. Scale bar, 20 µm.

Development • Supplementary information

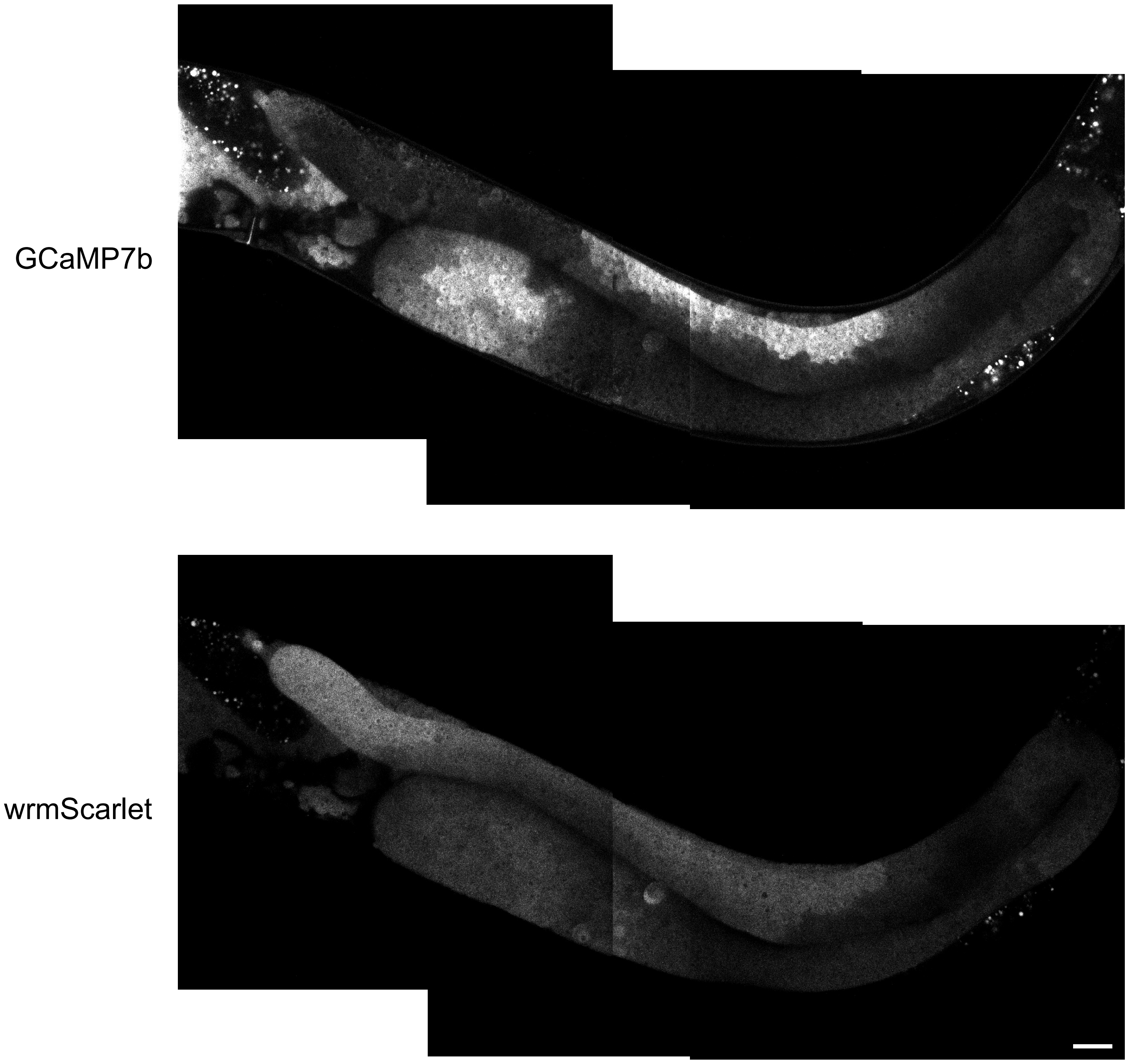

Fig. S7. Single-channel grayscale images of GCaMP7b and wrmScarlet for the overlaid compound image in Figure 5A. The focal plane varies slightly between the three individual images of the compound image. Scale bar, 20 µm.

Development • Supplementary information

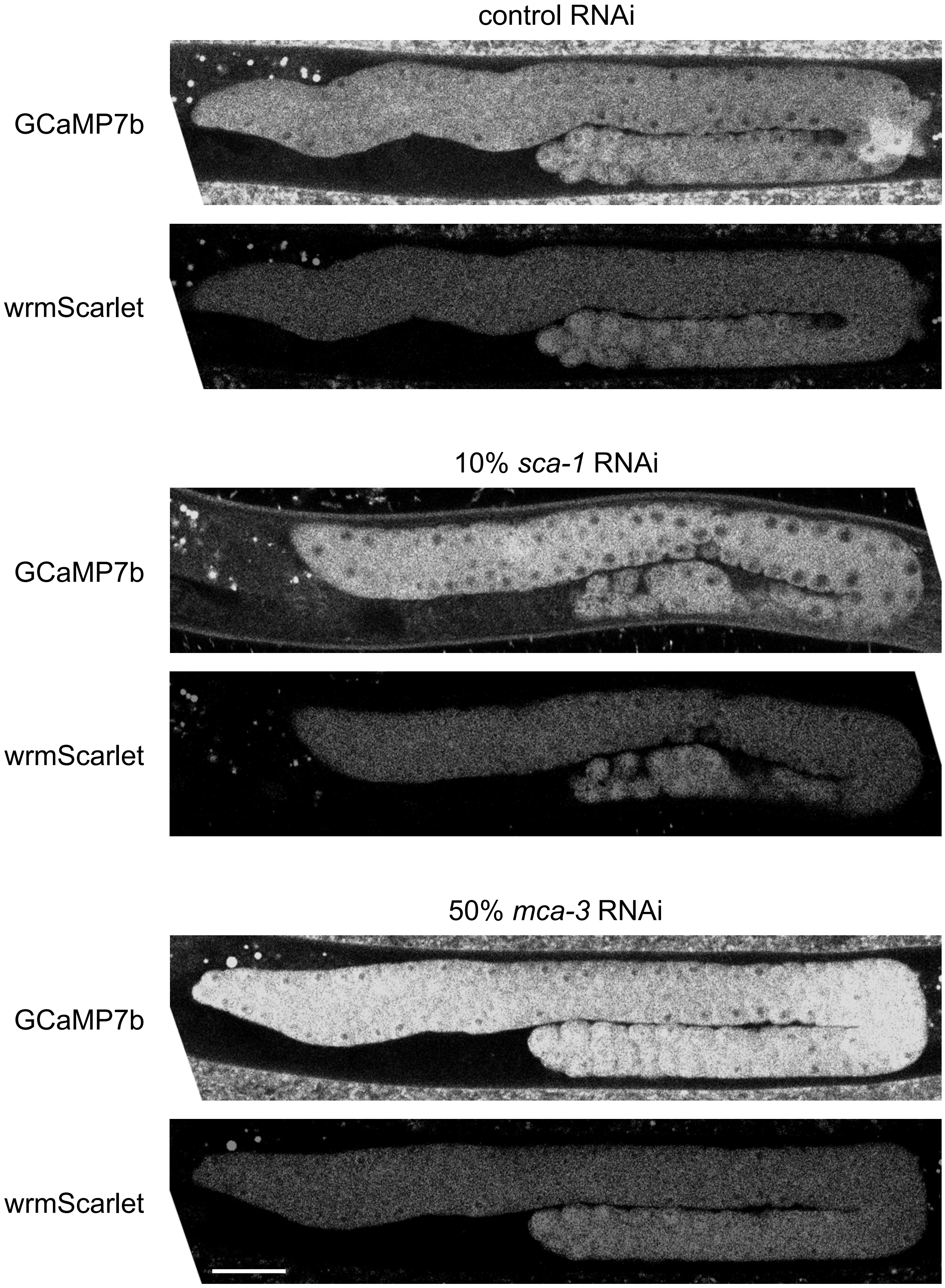

Fig. S8. Single-channel grayscale images of GCaMP7b and wrmScarlet for the overlaid images in Figure 7A. Scale bar, 20 μm.

Development • Supplementary information

Table S1. Strains list.

Strain	Genotype	Source
N2	wild-type Bristol strain	*Caenorhabditis* Genetics Center
WBM111 9	*wbmIs60*[*pie-1p*::3XFLAG::*dpy-10* crRNA::*unc-54* 3'UTR] (III:7007600)	*Caenorhabditis* Genetics Center
DCL569	*rde-1*(*mkc36*) V; *mkcSi13*[*sun-1p*::*rde-1*::*sun-1* 3'UTR + *unc-119*(+)] II	*Caenorhabditis* Genetics Center
JH3269	*pgl-1*(*ax3122*[*pgl-1*::gfp]) IV	*Caenorhabditis* Genetics Center
AZ212	*unc-119*(*ed3*) *ruIs32*[*pie-1p*::GFP::histoneH2B] III	*Caenorhabditis* Genetics Center
ET656	*ekIs32*[*pie-1p*::3XFLAG::GCaMP7b::*wrmScarlet*::*unc-54* 3'UTR]	This study
ET666	*folr-1*(*ek44*) X; *ekIs32* III	This study
ET670	*cki-2*(*ok2105*) II; *daf-16(mu86)* I; *ekIs32* III	This study
ET678	*gon-2*(*q388*)ts I; *ekIs32* III	This study
ET705	*rde-1(mkc36); mkcSi13[sun-1p::rde-1::sun-1 3'UTR + unc-119(+)] II; ekIs32*	This study
ET708	*ins-3*(*ok2488*) II; *ekIs32* III	This study
ET709	*daf-1*(*m40*ts) IV; *ekIs3* III	This study
ET715	*fog-1*(*q325*)/*hT2* I; *ekIs32* III	This study
ET718	*fem-3*(*q96ts*); *ekIs32* III	This study
ET719	*fem-1*(*hc17ts*); *him-5*(*e1490*) *dpy-21*(*e428*); *ekIs32*	This study
ET720	*pgl-1*(*ax3122*[*pgl-1*::gfp]) IV; *rde-1*(*mkc36*); *mkcSi13*[*sun-1p*::*rde-1*::*sun-1* 3'UTR + *unc-119*(+)] II	This study
ET721	*mog-5*(*q449*) *unc-4*(*e120*)/*mIn1* [*dpy-10*(*e128*)] II; *ekIs32* III	This study
ET722	*rde-1*(*mkc36*); *mkcSi13*[*sun-1p*::*rde-1*::*sun-1* 3'UTR + *unc-119*(+)] II; *pgl-1*(*ax3122*[*pgl-1*::gfp]) IV	This study
ET723	*daf-1*(*m40ts*) IV; *unc-119*(*ed3*) *ruIs32*[*pie-1*p::GFP:: histoneH2B] III	This study
EJ938	*gon-2*(*q388*) I; *dxIs1*[*gon-2p*::GON-2::GFP]	the kind gift of Eric Lambie
