# Supplementary material for: Basal cytoplasmic calcium levels regulate C. elegans germ stem cell proliferation
Source: Development. 2026 Jul 8;153(13):dev205301. doi: 10.1242/dev.205301 (PMC13380967; doi:10.1242/dev.205301)
Supplement: Supplementary file 1 [file develop-153-205301_review_history.pdf]

Basal cytoplasmic calcium levels regulate *C. elegans* germ stem cell proliferation

Fariha J. Khan, Parva M. Vyas, Aahana N. Shankaran, Alexandra C. Wells, Corrissa J. Velder, Rabia N. Kaya, Anuj R. Nagula and Edward T. Kipreos

DOI: 10.1242/dev.205301

Editor: Swathi Arur

Review timeline

Original submission:	14 October 2025
Editorial decision:	10 November 2025
First revision received:	30 April 2026
Accepted:	8 June 2026

Original submission

First decision letter

MS ID#: dev.205301

MS TITLE: Basal cytoplasmic calcium levels regulate C. elegans germ stem cell proliferation

AUTHORS: Edward T Kipreos; Alexandra C Wells; Parva M Vyas; Aahana N Shankaran; Corrissa J Velder; Rabia N Kaya

Dear Dr Kipreos,

I have now received all the referees' reports on the above manuscript, and have reached a decision. The referees' comments are appended below.

As you will see, the referees express considerable interest in your work, but provide critical recommendations to improve the rigor and conclusions in the manuscript, which include textual changes and experiments to obtain additional measures of proliferation, including measurements of G1 and S phase length, rather than rely only on M phase index, which has some biases, to fortify the core conclusions of the paper. If you are able to revise the manuscript along the lines suggested, which involve further experiments, I will be happy receive a revised version of the manuscript. Your revised paper will be re-reviewed by one or more of the original referees. Please also note that Development will normally permit only one round of major revision.

Please attend to all of the reviewers' comments and ensure that you upload both a 'clean' version of your Word file, along with a highlighted version clearly showing where you have made changes in the revised manuscript. Please avoid using 'Tracked changes' in Word files as these are lost in PDF conversion. I should be grateful if you would also provide a point-by-point response detailing how you have dealt with the points raised by the reviewers in the 'Response to Reviewers' box. If you do not agree with any of their criticisms or suggestions please explain clearly why this is so.

Reviewer 1

SUMMARY OF THE ADVANCE MADE IN THIS PAPER AND ITS POTENTIAL SIGNIFICANCE TO THE FIELD

© 2026. Published by The Company of Biologists under the terms of the Creative Commons Attribution License (https://creativecommons.org/licenses/by/4.0/).

In this manuscript, Wells et al, describe the role of Ca2+ levels in affecting stem cell proliferation in C. elegans. This work is built on the use of a powerful reagent they generated where they can visualize and measure Ca2+ levels in the C. elegans germ line. The data they present is very interesting and robust. They first show that reduced proliferation correlates with lower Ca2+ levels, and that increased proliferation correlates with increased Ca2+ levels. To move beyond correlation, they demonstrate that lowering Ca2+ levels results in reduced proliferation, and increasing Ca2+ levels increases proliferation. Importantly, for most of these manipulations they used more than one way of lowering or increasing proliferation or calcium levels. There are many different mechanisms that have been shown to be involved in the regulation of stem cell proliferation in the C. elegans germ line. These authors appear to have identified an additional regulatory mechanism.

SUGGESTIONS TO AUTHORS

1-In the introduction the authors describe the germline in C. elegans as being a syncytium. I believe that most people in the field refer to it as a 'partial syncytium' because there are membranes that partially surround the nuclei.
2-In the first section of the results section, the authors should provide a brief description (one or two sentences) on how GCaMp7b functions as a reporter of Ca2+ levels.
3-In the second section of the results section, the authors describe the results in figure 2. It is very striking that a group of cells has significantly higher Ca2+ levels at stages L4.5 to L4.9. It is also very interesting that their location is near the loop region. The authors do not comment on how common it is to see this pattern. Does this occur in all gonads observed? Is it always the same number of cells that are showing this higher level of Ca2+? Are they always in the same region? Some quantification on these questions would be beneficial. Additionally, there is no real discussion on why these cells may have higher Ca2+ levels, other than that they are located in the region where cells are likely transitioning from spermatogenesis to oogenesis. Given that these cells are not proliferative, but the focus of the paper is primarily on the role of Ca2+ in proliferation, the authors may want to provide more explanation/speculation on what may be happening here.
4-In figure 2c it appears as though Ca2+ levels are higher in the distal arm than in the proximal arm. Likewise, in 1D and 1E there may be slightly higher levels at the end of the proliferative zone. However, it is difficult to tell if this is something that is common in all gonads, or simply random variation. It is intriguing that there may be slightly higher levels in the region where cells are transitioning from mitosis to meiosis. Can the authors provide relative expression data across the length of the distal arm of the gonad? Since this paper is focused on Ca2+ levels regulating stem cell proliferation, it seems that accurately measuring Ca2+ levels in the region of the gonad containing stem cells vs. regions of the gonad that are post-mitotic would be highly beneficial.
5-The authors comment, with respect to Fig 2G-H, that 'The switch from only a few germ cells with very elevated Ca2+ to more cells with less elevated Ca2+ levels occurs in the region that gives rise to oocytes, which also have elevated Ca2+ levels'. As the authors are referring to elevated Ca2+ in various regions, it would be of benefit to know how elevated they are, in which regions, and how variable this is across gonads. Therefore, as stated above, it would be beneficial to have measurements across the length of the gonad.
6-Figure 4A could benefit from a DAPI panel so that the extent of the tumor can be compared to the Ca2+ levels.
7-Figure 5 is labelled awkwardly. 5A should be first in the figure. The authors may want to consider re-labeling the panels.
8-The section of the results section that describes germ cell numbers in gon-2 animals vs. wt seems to suggest that different regions of the gonad were counted in wt and mutant. In gon-2 the authors counted cells in the entire region of the germ line that lies along the dorsal side, whereas in wt animals they counted the total number of cells in the mitotic zone. If the authors are to compare the number of cells in each, and perform a statistical test as to the significance, they should count equivalent regions in the gonad.
9-In the third section of the results section (Figure 3), the authors state that ins-3 animals have 24.5% reduced Ca2+ levels relative to wild-type. The authors should state the region of the gonad where they are taking these measurements, and whether the reduction is found throughout the gonad.
10-The authors utilize a number of different methods to reduce proliferation in the germline, which resulted in lower Ca2+ levels, and other methods to increase proliferation (proximal tumors), resulting in increased Ca2+ levels. It is interesting that the authors did not use the most direct method of decreasing and increasing proliferation of the stem cells- that of modulating GLP-

© 2026. Published by The Company of Biologists under the terms of the Creative Commons Attribution License (https://creativecommons.org/licenses/by/4.0/).

1/Notch signaling. The temperature sensitive glp-1(bn18) allele has reduced proliferation in the distal end, while the glp-1 gain of function alleles, ar202 and oz264, have increased proliferation in the distal end, as well as some proximal proliferation. There can be many reasons for proximal proliferation to occur, including de-differentiation. Therefore, it is difficult to interpret why Ca2+ levels are elevated in these tumors. However, glp-1(gf) distal over-proliferation is thought to truly be the result of increased stem cell proliferation. Is there a reason why the authors did not use the glp-1 alleles?

Reviewer 2

Wells et al. investigated calcium signaling in germline development and a possible role in germ cell proliferation. They generate a ratiometric GCaMP7b germline specifically expressed reporter and identify differences in calcium levels between the L4 and adult stages and between different regions in the germline. Wells et al. identified GON-2 as a calcium channel that functions in the maintenance of physiological levels of calcium in the germline. Finally, the authors present data suggesting that basal calcium levels may control proliferation rate in germ cells and/or that germ cell proliferation rate may control basal calcium levels. This work has the potential to make a novel connection between cell cycle control and cellular calcium levels.

A major conclusion of the manuscript is that there is a correlation between calcium level and proliferation rate; elevated calcium levels are associated with increased proliferation and lower calcium levels are associated with decreased proliferation, where proliferation rate in essential all of the examples cited and the experiments performed was defined by mitotic index (# of M-phase/total relevant cell population). However, mitotic index is an imperfect measure of proliferation rate/cell cycle length, at least in part because it is short in duration relative to the remaining cell cycle stages. Furthermore, below are two examples where M phase index gives the wrong answer concerning a possible change in proliferation rate. Wang et al., 2020 (PMID: 32804074) confirmed that fbf-1 mutants had an increased M phase index relative to wild type (54%) that was reported to result in a faster cell cycle. However, when they measure the lengths of G2 and S-phase, they were found to be the same as wild type, resulting in no change in cell cycle length (G1 length is vanishingly small in the C. elegans germline, Lara-Gonzalez et al. 2019 (PMID: 31588029)). Kocsisova et al., 2019 (PMID: 30936182) investigated how proliferation changes in stem and progenitor germ cells with age, measuring the M phase index and length of G2 and S phases. They found that while the M phase index did not change, the S and G2 phases almost doubled in duration in older animals. Given the uncertainty of the effect of the M phase index on cell cycle length, the authors should minimally determine the lengths of G2 and S phases for the gon-2 mutant and sca-1 RNAi or mca-3 RNAi, using the methodology of Wang et al., 2020 (PMID: 32804074) and Kocsisova et al., 2018 (PMID: 30394383). Solidifying the cycle stage(s) that is associated with changes in calcium level will increase the impact of the study and begin to connect this work with the many cell cycle studies in animals.

The authors indicate that proximal tumors in cki-2(ok2105); daf-16(mu86) have increased proliferation rate. Increased compared to what, given that these are ectopically proliferating germ cells? Please provide a reference for cki-2(ok2105); daf-16(mu86) causing proximal tumors.

The manuscript does not describe how the mitotic index values shown in Figures 5D and 6D were determined.

Reviewer 3

SUMMARY OF THE ADVANCE MADE IN THIS PAPER AND ITS POTENTIAL SIGNIFICANCE TO THE FIELD

This manuscript introduces a ratiometric calcium reporter for Ca++ levels in C. elegans, that should be of significant interest and use to the community. Analysis of Ca++ levels in the germ line suggests that high Ca++ levels may be an early, if not the earliest, marker for the oocyte fate. Further, high Ca++ levels are seen in proliferating germ cells. For the latter observation, the possibility that Ca++ levels may be instructive for proliferation is supported by experiments that alter Ca++ transport. Overall, the work is novel, interesting, and generally well presented.

SUGGESTIONS TO AUTHORS

© 2026. Published by The Company of Biologists under the terms of the Creative Commons Attribution License (https://creativecommons.org/licenses/by/4.0/).

1. The oocyte results observation is given less attention, though it is very exciting; the idea that Ca++ cells have adopted the oocyte fate would further benefit from the examination of L4 and adult mutants that produce only oocytes or no oocytes (fem/fog and mog, respectively) to demonstrate correlation of elevated Ca++ and the early oocyte fate.
2. Several important points in the introduction require references, correction and/or clarification.
A. "Once these germ cells enter meiosis, they begin a regulatory program to become oocytes". This is incorrect. The earliest germ cells undergo meiosis and become sperm. There is a long prophase of meiosis I for oocytes that does not occur for spermatogenic germ cells. Perhaps that is what is meant?
B. "they respond by increasing their rate of germ cell proliferation to rapidly produce progeny." This statement requires a reference. If the point is that they rapidly increase the number of germ cells after starvation, references are as for ARD (Seidel and Kimble 2015 and Roy et al., 2016), both of which measured mitotic index and cell cycle stages over time during recovery.
C. "Nematodes that can generate progeny faster have a clear evolutionary advantage." This statement requires a reference; generating more progeny does not necessarily support evolutionary advantage, especially in cases where progeny compete for resources (see Hughes et al., 2007 for example).
D. "availability of food through insulin signaling via specific insulin ligands (INS-3 and INS-33)" This has not been tested directly.
E. "Loss of these signaling pathways cause a decrease in germ cell proliferation…" Though loss of these pathways result in a smaller pool of proliferating germ cells, regarding insulin and TOR signaling, Michaelson et al., and Korta et al report a decrease in mitotic index in larval germ cells, but not in adults, which cycle slower than larval germ cells (see also Roy et al., 2016). TGF-b, by contrast, affects Notch signaling (see Pekar et al., 2017), and the mutant causes germ cells to differentiate closer to the distal end, but does not change the rate of proliferation.
F. "and starvation causes a cessation of proliferation." This statement requires a new reference. Moreover, cessation of proliferation is only the case for starvation conditions, not the sub-starvation conditions reported in the referenced papers.
3. "TGF-b signaling is also known to promote germ cell proliferation (Dalfo et al., 2012)." See also Pekar et al., 2017. In the case of TGF-b, main effect is not via cell cycle. While the number of germ cells is reduced, this reduction is likely due to differentiation, not cell cycle inhibition.
4. It is unclear to the reader how the authors arrived at the various percentages of RNAi bacterial dilutions that were used in the experiments presented in Figure 6. Perhaps an explanatory sentence would help in the results of in the methods.
5. "Our data on starvation suggests that reductions in Ca2+ levels do not directly follow from a reduction in the rate of proliferation…" While the point about other cues is well taken, it may be worth considering that larval germ cells proliferate faster than adult germ cells.
6. "Therefore, the differential reductions in basal Ca^{2+} levels between the two developmental stages do not correlate with their relative changes in proliferation." In general, it is important to distinguish between "proliferation" and "proliferation rate", which, in this case could also be referred to as Mitotic index, since that is what was actually measured.
7. The authors may wish to cite WormBase: https://doi.org/10.1093/genetics/iyae050

First revision

Author response to reviewers' comments

Reviewer 1: SUMMARY OF THE ADVANCE MADE IN THIS PAPER AND ITS POTENTIAL SIGNIFICANCE TO THE FIELD

In this manuscript, Wells et al, describe the role of Ca2+ levels in affecting stem cell proliferation in C. elegans. This work is built on the use of a powerful reagent they generated where they can visualize and measure Ca2+ levels in the C. elegans germ line. The data they present is very interesting and robust. They first show that reduced proliferation correlates with lower Ca2+ levels, and that increased proliferation correlates with increased Ca2+ levels. To move beyond correlation, they demonstrate that lowering Ca2+ levels results in reduced

© 2026. Published by The Company of Biologists under the terms of the Creative Commons Attribution License (https://creativecommons.org/licenses/by/4.0/).

proliferation, and increasing Ca2+ levels increases proliferation. Importantly, for most of these manipulations they used more than one way of lowering or increasing proliferation or calcium levels. There are many different mechanisms that have been shown to be involved in the regulation of stem cell proliferation in the C. elegans germ line. These authors appear to have identified an additional regulatory mechanism.

SUGGESTIONS TO AUTHORS

1-In the introduction the authors describe the germline in C. elegans as being a syncytium. I believe that most people in the field refer to it as a 'partial syncytium' because there are membranes that partially surround the nuclei.

> We have corrected the wording to reflect that the germline is a partial syncytium.

2-In the first section of the results section, the authors should provide a brief description (one or two sentences) on how GCaMp7b functions as a reporter of Ca2+ levels.

> We have reorganized the first two paragraphs of the results section to introduce earlier how the combination of GCaMP7b and wrmScarlet produces a ratiometric reporter of Ca^{2+} levels.

3-In the second section of the results section, the authors describe the results in figure 2. It is very striking that a group of cells has significantly higher Ca2+ levels at stages L4.5 to L4.9. It is also very interesting that their location is near the loop region. The authors do not comment on how common it is to see this pattern. Does this occur in all gonads observed? Is it always the same number of cells that are showing this higher level of Ca2+? Are they always in the same region? Some quantification on these questions would be beneficial. Additionally, there is no real discussion on why these cells may have higher Ca2+ levels, other than that they are located in the region where cells are likely transitioning from spermatogenesis to oogenesis. Given that these cells are not proliferative, but the focus of the paper is primarily on the role of Ca2+ in proliferation, the authors may want to provide more explanation/speculation on what may be happening here.

> We now provide more insights into the subset of L4-stage germ cells that have elevated Ca^{2+} levels. In Fig. S3A, we present the distribution in the number of elevated Ca^{2+} cells in the L4.3 to L4.9 sub-stages. This shows that the elevated Ca^{2+} cells start to appear in the L4.4 sub-stage, and appear to peak around sub-stage L4.7. The distribution suggests that most (if not all) gonads probably have elevated-Ca^{2+} cells at some point during L4, but their presence is shorter than the ~4.7 hr period between L4.4 and L4.7 sub-stages, so a percentage of gonads do not have expression during this period, although L4.7 gonads examined did have these cells (8/8). In Fig. S3B, we show that the GCaMP7b/wrmScarlet ratio is approximately 2.7-fold higher in the elevated-Ca^{2+} cells relative to the surrounding cells. In Fig. S3C, we show that relative to the midpoint of the gonad arm bend, the elevated-Ca^{2+} germ cells start off proximal to the bend in sub-stage L4.4 and end up with a relatively equal distribution around the bend in L4.9. Based on the location of these cells between the proximal spermatogenic program and the distal oogenetic program (as well as the effects of sex determination changes, which are discussed below), we propose in the Discussion that the elevated-Ca^{2+} cells arise in response to the spermatogenesis-to-oogenesis transition and/or the interplay between the spermatogenic and oogenic programs within the cells.

4-In figure 2c it appears as though Ca2+ levels are higher in the distal arm than in the proximal arm. Likewise, in 1D and 1E there may be slightly higher levels at the end of the proliferative zone. However, it is difficult to tell if this is something that is common in all gonads, or simply random variation. It is intriguing that there may be slightly higher levels in the region where cells are transitioning from mitosis to meiosis. Can the authors provide relative expression data across the length of the distal arm of the gonad? Since this paper is

© 2026. Published by The Company of Biologists under the terms of the Creative Commons Attribution License (https://creativecommons.org/licenses/by/4.0/).

focused on Ca2+ levels regulating stem cell proliferation, it seems that accurately measuring Ca2+ levels in the region of the gonad containing stem cells vs. regions of the gonad that are post-mitotic would be highly beneficial.

We now provide this information in a new Fig. 3. Line graphs of the GCaMP7b/wrmScarlet ratio from the distal end of the gonad to the proximal end of the gonad are shown for L4.3–L4.5 and L4.6–L4.9 sub-stages, and young adults. In the L4.6–L4.9 larvae and young adults, we see a significant increase in the GCaMP7b/wrmScarlet ratio in the distal region of the germline that includes the mitotic proliferation zone. This provides additional evidence that increased levels of Ca^{2+} are linked to proliferation.

5-The authors comment, with respect to Fig 2G-H, that 'The switch from only a few germ cells with very elevated Ca2+ to more cells with less elevated Ca2+ levels occurs in the region that gives rise to oocytes, which also have elevated Ca2+ levels'. As the authors are referring to elevated Ca2+ in various regions, it would be of benefit to know how elevated they are, in which regions, and how variable this is across gonads. Therefore, as stated above, it would be beneficial to have measurements across the length of the gonad.

The distal-to-proximal line graphs of Fig. 3C show the oocyte region in young adults has an ~50% increase in the GCaMP7b/wrmScarlet ratio. This graph also demonstrates that, as described, the proximal spermatocyte region has a significant decrease in the GCaMP7b/wrmScarlet ratio.

6-Figure 4A could benefit from a DAPI panel so that the extent of the tumor can be compared to the Ca2+ levels.

It is technically difficult to co-stain with DAPI (which requires fixation) and also observe the GCaMP7b signal (which requires Ca^{2+} in live cells). Zooming in on the (now Fig. 5A) image reveals individual cells (GCaMP7b is partially excluded from nuclei, which delineates cells). To clarify the extent of the tumor, we have marked it on the image. We notice that the pattern of the distribution of elevated Ca^{2+} signal sometimes varies within these older (two-day-old adult) *cki-2*; *daf-16* tumors, but the overall Ca^{2+} level is significantly higher in the tumor than in the wild-type distal mitotic zone. We also mention that one-day-old adult tumors have mitotic cells that are more evenly distributed within the tumor, with a somewhat more uniform elevated Ca^{2+} signal. We now provide data showing that the mitotic index (and Ca^{2+} levels) are higher in the one-day-old adult *cki-2*; *daf-16* tumors compared to the distal mitotic zone of one-day-old wild-type adults.

7-Figure 5 is labelled awkwardly. 5A should be first in the figure. The authors may want to consider re-labeling the panels.

We have revised the layout of the figure (now Fig. 6) to make panel A the first panel in the figure.

8-The section of the results section that describes germ cell numbers in gon-2 animals vs. wt seems to suggest that different regions of the gonad were counted in wt and mutant. In gon-2 the authors counted cells in the entire region of the germ line that lies along the dorsal side, whereas in wt animals they counted the total number of cells in the mitotic zone. If the authors are to compare the number of cells in each, and perform a statistical test as to the significance, they should count equivalent regions in the gonad.

We agree that the comparison of the germ cells on the distal section of the *gon-2* mutant germline vs. the mitotic zone of wild-type was not an equivalent comparison. We now report the average number of germ cells in the distal section of wild-type L4, and as expected, the number is significantly greater than the number of germ cells in the mitotic zone. This direct comparison allows us to conclude that there are almost 6-fold more germ cells in the

© 2026. Published by The Company of Biologists under the terms of the Creative Commons Attribution License (https://creativecommons.org/licenses/by/4.0/).

distal section of the wild-type germline compared to the same region in *gon-2* mutants.

9-In the third section of the results section (Figure 3), the authors state that ins-3 animals have 24.5% reduced Ca2+ levels relative to wild-type. The authors should state the region of the gonad where they are taking these measurements, and whether the reduction is found throughout the gonad.

We now clarify in the text that the comparison between *ins-3* and wild type Ca^{2+} levels is in the distal mitotic zone. We observe a reduction in Ca^{2+} level over the full gonad (not just in the mitotic zone). We now present a Fig. S4 that shows that the reduction in Ca^{2+} level extends beyond the distal mitotic zone.

10-The authors utilize a number of different methods to reduce proliferation in the germline, which resulted in lower Ca2+ levels, and other methods to increase proliferation (proximal tumors), resulting in increased Ca2+ levels. It is interesting that the authors did not use the most direct method of decreasing and increasing proliferation of the stem cells- that of modulating GLP-1/Notch signaling. The temperature sensitive glp-1(bn18) allele has reduced proliferation in the distal end, while the glp-1 gain of function alleles, ar202 and oz264, have increased proliferation in the distal end, as well as some proximal proliferation. There can be many reasons for proximal proliferation to occur, including de-differentiation. Therefore, it is difficult to interpret why Ca2+ levels are elevated in these tumors. However, glp-1(gf) distal over-proliferation is thought to truly be the result of increased stem cell proliferation. Is there a reason why the authors did not use the glp-1 alleles?

We did not use *glp-1* alleles because the *glp-1* locus and the SKI LODGE *pie-1p* insertion site for GCaMP7b::P2A::wrmScarlet are close together in the central region of chromosome III. Therefore, it would be technically difficult to combine the transgene and *glp-1* gain-of-function alleles. We performed *glp-1* RNAi to assess the loss-of-function phenotype. However, the germ cells in L4-stage *glp-1* RNAi animals had already entered meiosis, and there was no GCaMP7b or wrmScarlet signal above background levels. It is known that mature sperm are transcriptionally silent (Shakes et al., 2009, PLOS Genetics 5: e1000611), and this is likely the reason that there is a lack of expression of GCaMP7b::P2A::wrmScarlet in the *glp-1* RNAi gonads.

Reviewer 2: Wells et al. investigated calcium signaling in germline development and a possible role in germ cell proliferation. They generate a ratiometric GCaMP7b germline specifically expressed reporter and identify differences in calcium levels between the L4 and adult stages and between different regions in the germline. Wells et al. identified GON-2 as a calcium channel that functions in the maintenance of physiological levels of calcium in the germline. Finally, the authors present data suggesting that basal calcium levels may control proliferation rate in germ cells and/or that germ cell proliferation rate may control basal calcium levels. This work has the potential to make a novel connection between cell cycle control and cellular calcium levels.

A major conclusion of the manuscript is that there is a correlation between calcium level and proliferation rate; elevated calcium levels are associated with increased proliferation and lower calcium levels are associated with decreased proliferation, where proliferation rate in essential all of the examples cited and the experiments performed was defined by mitotic index (# of M-phase/total relevant cell population). However, mitotic index is an imperfect measure of proliferation rate/cell cycle length, at least in part because it is short in duration relative to the remaining cell cycle stages. Furthermore, below are two examples where M phase index gives the wrong answer concerning a possible change in proliferation rate. Wang et al., 2020 (PMID: 32804074) confirmed that fbf-1 mutants had an increased M phase index relative to wild type (54%) that was reported to result in a faster cell cycle. However, when they measure the lengths of G2 and S-phase, they were found to be the same as wild type, resulting in no change in cell cycle length (G1 length is

© 2026. Published by The Company of Biologists under the terms of the Creative Commons Attribution License (https://creativecommons.org/licenses/by/4.0/).

vanishingly small in the C. elegans germline, Lara-Gonzalez et al. 2019 (PMID: 31588029)). Kocsisova et al., 2019 (PMID: 30936182) investigated how proliferation changes in stem and progenitor germ cells with age, measuring the M phase index and length of G2 and S phases. They found that while the M phase index did not change, the S and G2 phases almost doubled in duration in older animals. Given the uncertainty of the effect of the M phase index on cell cycle length, the authors should minimally determine the lengths of G2 and S phases for the gon-2 mutant and sca-1 RNAi or mca-3 RNAi, using the methodology of Wang et al., 2020 (PMID: 32804074) and Kocsisova et al., 2018 (PMID: 30394383). Solidifying the cycle stage(s) that is associated with changes in calcium level will increase the impact of the study and begin to connect this work with the many cell cycle studies in animals.

The reviewer correctly points out that relying on mitotic index alone may not accurately reflect the effect on proliferation rate. The reviewer suggested that we analyze cell-cycle stage lengths in L4-stage animals (the stage at which we focused most of our analyses). However, we chose an alternate method to test whether germ-cell proliferation was increased: direct measurement of germ-cell accumulation in early larvae by counting germ-cell numbers at a set time after release of L1-stage arrest.

We chose this approach for two reasons. First, the EdU-based cell-cycle methods used by Wang et al. (2020) and Kocsisova et al. (2018) require robust staining of progenitor-zone cells with anti-REC-8 or anti-WAPL-1 antibodies. These rabbit polyclonal antibodies were generated through the modENCODE project and then distributed by Novus Biologicals. However, both reagents have sold out and are no longer commercially available. Thus, the suggested approach was not technically feasible for us.

Second, direct germ-cell counts provide a more direct measure of proliferation than inferring proliferative rate from cell-cycle phase distributions in the L4 germline. At later larval stages, germ cells are both cycling and entering meiosis, so the proliferative zone is not a closed population. The Wang and Kocsisova methods address this by measuring meiotic entry separately, but the calculation of cell cycle length still requires inference from a dynamically changing population. In contrast, counting germ cell numbers provides a direct measure of proliferation in early larvae before meiotic entry, thereby avoiding the complication of cells leaving the cell cycle. This approach is conceptually similar to the early larval germ-cell doubling-time assay used by Wang et al. (2020), in which germ-cell numbers were counted directly before meiotic differentiation.

We now show that germline-specific RNAi inactivation of *sca-1* and *mca-3* increases Ca^{2+} levels in early larvae, and this is associated with increased germ cell numbers in early larvae (germ cell counts were made 24 hrs after placing starvation-arrested L1 larvae onto food/bacteria) (Fig. 7E–H).

The authors indicate that proximal tumors in cki-2(ok2105); daf-16(mu86) have increased proliferation rate. Increased compared to what, given that these are ectopically proliferating germ cells? Please provide a reference for cki-2(ok2105); daf-16(mu86) causing proximal tumors.

Previously, we utilized a strain with *cki-2*(*ok2105*); *daf-16*(*mu86*); and *glp-1*(*ar202ts*) mutations to isolate germ cells from animals with large tumors that developed at 25°C (while the animals could be maintained without tumors at 16°C). We now cite that use in the manuscript. We subsequently found that *cki-2*; *daf-16* mutations together produce proximal tumors at 25°C. This result has not been published previously.

To demonstrate that the proliferation rate is increased in these mutants, we now show that the germline of one-day old adult *cki-2*; *daf-16* mutants at 25°C have an increased mitotic index relative to that of the mitotic zone in

© 2026. Published by The Company of Biologists under the terms of the Creative Commons Attribution License (https://creativecommons.org/licenses/by/4.0/).

one-day old wild-type adults at 25°C. We also show that the Ca^{2+} level is higher in the one-day-old adult *cki-2*; *daf-16* proximal tumors. These are shown in the new Fig. 5C,D.

The manuscript does not describe how the mitotic index values shown in Figures 5D and 6D were determined.

We have added text to the Microscopy and Image Analysis section of the Methods that describes how the mitotic index was experimentally obtained and calculated.

Reviewer 3: SUMMARY OF THE ADVANCE MADE IN THIS PAPER AND ITS POTENTIAL SIGNIFICANCE TO THE FIELD

This manuscript introduces a ratiometric calcium reporter for Ca++ levels in C. elegans, that should be of significant interest and use to the community. Analysis of Ca++ levels in the germ line suggests that high Ca++ levels may be an early, if not the earliest, marker for the oocyte fate. Further, high Ca++ levels are seen in proliferating germ cells. For the latter observation, the possibility that Ca++ levels may be instructive for proliferation is supported by experiments that alter Ca++ transport. Overall, the work is novel, interesting, and generally well presented.

SUGGESTIONS TO AUTHORS

1. The oocyte results observation is given less attention, though it is very exciting; the idea that Ca++ cells have adopted the oocyte fate would further benefit from the examination of L4 and adult mutants that produce only oocytes or no oocytes (fem/fog and mog, respectively) to demonstrate correlation of elevated Ca++ and the early oocyte fate.

We have now performed the suggested experiments. We observed that feminization of the germline with *fem-1* or *fog-1* loss-of-function mutants abolished the presence of the elevated-Ca^{2+} cells. Conversely, masculinization of the germline with Mog mutants (*fem-1* gain-of-function and *mog-5* loss-of-function) significantly reduced the presence of the elevated-Ca^{2+} cells. This data is presented in a new Table 1. Notably, the elevated-Ca^{2+} cells arise between the spermatogenic and oogenic programs in mid-to-late L4-stage larvae, at a time when the oogenic program has initiated and the spermatogenic program is still operational. Based on these results, we propose that the spermatogenesis-to-oogenesis transition or the interplay between these two programs within specific germ cells leads to the elevated Ca^{2+} levels.

2. Several important points in the introduction require references, correction and/or clarification.

A. "Once these germ cells enter meiosis, they begin a regulatory program to become oocytes". This is incorrect. The earliest germ cells undergo meiosis and become sperm. There is a long prophase of meiosis I for oocytes that does not occur for spermatogenic germ cells. Perhaps that is what is meant?

The cited sentence was a continuation of a prior sentence in which it was stated “In adult hermaphrodites, as germ cells leave the DTC-defined stem cell niche”, and thus we were referring to germ cells that would enter a differentiaton program to become oocytes. To clarify this for the reader, we changed the wording of the prior sentence to “In *older* adult hermaphrodites”, so there would be no confusion that spermatogenesis was still occurring. We also clarified the context of the cited sentence to “Once germ cells exit the DTC-defined stem cell niche, they enter meiosis and will begin the regulatory program for oocyte differentiation.”

© 2026. Published by The Company of Biologists under the terms of the Creative Commons Attribution License (https://creativecommons.org/licenses/by/4.0/).

B. "they respond by increasing their rate of germ cell proliferation to rapidly produce progeny." This statement requires a reference. If the point is that they rapidly increase the number of germ cells after starvation, references are as for ARD (Seidel and Kimble 2015 and Roy et al., 2016), both of which measured mitotic index and cell cycle stages over time during recovery.

We have added the suggested references, as well as Korta et al., 2012, which shows that reduced food levels significantly reduce the rate of germ cell proliferation. The Seidel and Kimble, 2015 paper also shows that re-feeding after starvation for 12 hrs increases germ cell proliferation, as this is a more transient food deprivation than the extended starvation in ARD.

C. "Nematodes that can generate progeny faster have a clear evolutionary advantage." This statement requires a reference; generating more progeny does not necessarily support evolutionary advantage, especially in cases where progeny compete for resources (see Hughes et al., 2007 for example).

We now have revised the sentence and section to emphasize that it is the rapidity of producing progeny that is advantageous for boom-bust life cycles, rather than the total number of progeny that are produced. We cite Hodgkin and Barnes, 1991, to support this.

D. "availability of food through insulin signaling via specific insulin ligands (INS-3 and INS-33)" This has not been tested directly.

We thank the reviewer for this correction. We revised the text to remove the unsupported statement that food availability acts through INS-3 and INS-33.

E. "Loss of these signaling pathways cause a decrease in germ cell proliferation…" Though loss of these pathways result in a smaller pool of proliferating germ cells, regarding insulin and TOR signaling, Michaelson et al., and Korta et al report a decrease in mitotic index in larval germ cells, but not in adults, which cycle slower than larval germ cells (see also Roy et al., 2016). TGF-b, by contrast, affects Notch signaling (see Pekar et al., 2017), and the mutant causes germ cells to differentiate closer to the distal end, but does not change the rate of proliferation.

We now clarify that insulin and TOR signaling affect larval proliferation. We have also extended this section to clarify that TGF-beta signaling is primarily thought to function through promoting the expression of LAG-2 in the DTC that signals through GLP-1 on germ cells to keep germ cells dividing mitotically. However, we also cite the Dalfo et al, 2012, result that TGF-beta signaling promotes germ cell proliferation in a GLP-1 independent manner (i.e., in *gld-2 gld-1*, *glp-1* mutant tumors that lack functional GLP-1).

F. "and starvation causes a cessation of proliferation." This statement requires a new reference. Moreover, cessation of proliferation is only the case for starvation conditions, not the sub-starvation conditions reported in the referenced papers.

We have revised the wording and references to indicate that dietary restriction and short-term food deprivation causes decreases in germ cell proliferation while extended starvation causes a cessation of proliferation (citing Korta et al., 212; Roy et al., 2016; and Seidel and Kimble, 2015).

3. "TGF-b signaling is also known to promote germ cell proliferation (Dalfo et al., 2012)." See also Pekar et al., 2017. In the case of TGF-b, main effect is not via cell cycle. While the number of germ cells is reduced, this reduction is likely due to differentiation, not cell cycle inhibition.

As discussed above, Dalfo et al., 2012, indicates that TGF-beta signaling can promote germ cell proliferation independently of the GLP-1 mediated block on differentiation. To extend this further, we now provide data that in the

© 2026. Published by The Company of Biologists under the terms of the Creative Commons Attribution License (https://creativecommons.org/licenses/by/4.0/).

early larvae (before germ cells enter meiosis), *daf-1* inactivation causes a reduction in germ cell numbers (during either 16 or 24 hrs post feeding of starvation-arrested L1 larvae). This supports the position that TGF-beta signaling also promotes germ cell proliferation.

4. It is unclear to the reader how the authors arrived at the various percentages of RNAi bacterial dilutions that were used in the experiments presented in Figure 6. Perhaps an explanatory sentence would help in the results of in the methods.

We have added to the RNAi section of Methods the explanation that full *sca-1* and *mca-3* RNAi treatments result in embryonic and larval arrest, and that the concentrations used were either those found to function without impairing development in other publications, or were the highest concentration tested that did not produce overt developmental delays.

5. "Our data on starvation suggests that reductions in Ca2+ levels do not directly follow from a reduction in the rate of proliferation…" While the point about other cues is well taken, it may be worth considering that larval germ cells proliferate faster than adult germ cells.

We have reworded this section to take into account the differences in germ cell proliferation rate between L4 larvae and adults. We now cite Roy et al., 2016 for this information.

6. "" developmental stages do not correlate with their relative changes in proliferation." In general, it is important to distinguish between "proliferation" and "proliferation rate", which, in this case could also be referred to as Mitotic index, since that is what was actually measured.

We thank the reviewer for this point. We now refer to this as "M-phase cells per mitotic zone", as that is what was measured in the Seidel and Kimble paper. This metric (which differs from mitotic index) also explains why the basal levels of M-phase cells per mitotic zone are roughly equivalent between L4 and adult stages in their paper, yet the proliferation rate is known to be higher in L4 larvae, presumably because there are more germ cells in the mitotic zone of adults (which would produce a lower mitotic index for the same number of M-phase cells).

7. The authors may wish to cite WormBase: https://doi.org/10.1093/genetics/iyae050

We thank the reviewer for the reference. We now cite WormBase in the Methods section.

<u>Second decision letter</u>

MS ID#: dev.205301R1

MS TITLE: Basal cytoplasmic calcium levels regulate C. elegans germ stem cell proliferation

AUTHORS: Edward T. Kipreos, Fariha J. Khan, Parva M. Vyas, Aahana N. Shankaran, Alexandra C. Wells, Corrissa J. Velder, Rabia N. Kaya and Anuj R. Nagula

Dear Dr Kipreos,

I am happy to tell you that your manuscript has been accepted for publication in Development, pending our standard publication integrity checks.

<u>Reviewer 1</u>

© 2026. Published by The Company of Biologists under the terms of the Creative Commons Attribution License (https://creativecommons.org/licenses/by/4.0/).

SUMMARY OF THE ADVANCE MADE IN THIS PAPER AND ITS POTENTIAL SIGNIFICANCE TO THE FIELD

As mentioned in my first review of this paper, the data in this paper is very interesting and robust, and will of interest as they have identified an additional mechanism involved in the control of stem cell proliferation. In my opinion, the authors have provided additional experimentation and explanation that has addressed the three reviewer's comments/suggestions.

SUGGESTIONS TO AUTHORS

In the results section, the authors state that 'TGF-B signaling is also known to promote the mitotic proliferation of germ cells both through increasing GLP-1 expression in the DTC…'. I believe that it is through increasing LAG-2 expression that this is accomplished.

Reviewer 2

SUGGESTIONS TO AUTHORS

The authors have addressed the concerns of this reviewer.

© 2026. Published by The Company of Biologists under the terms of the Creative Commons Attribution License (https://creativecommons.org/licenses/by/4.0/).
